# Association between diabetes mellitus and active tuberculosis: A systematic review and meta-analysis

**DOI:** 10.1371/journal.pone.0187967

**Published:** 2017-11-21

**Authors:** Rami H. Al-Rifai, Fiona Pearson, Julia A. Critchley, Laith J. Abu-Raddad

**Affiliations:** 1 Infectious Disease Epidemiology Group, Weill Cornell Medical College–Qatar, Cornell University, Qatar Foundation–Education City, Doha, Qatar; 2 Department of Healthcare Policy and Research, Weill Cornell Medical College, Cornell University, New York, New York, United States of America; 3 Institute of Public Health, College of Medicine and Health Sciences, United Arab Emirates University, Al-Ain, United Arab Emirates; 4 Population Health Research Institute, St George’s, University of London, London, United Kingdom; 5 College of Public Health, Hamad bin Khalifa University, Qatar Foundation, Education City, Doha, Qatar; Universidad Miguel Hernandez de Elche, SPAIN

## Abstract

The burgeoning epidemic of diabetes mellitus (DM) is one of the major global health challenges. We systematically reviewed the published literature to provide a summary estimate of the association between DM and active tuberculosis (TB). We searched Medline and EMBASE databases for studies reporting adjusted estimates on the TB–DM association published before December 22, 2015, with no restrictions on region and language. In the meta-analysis, adjusted estimates were pooled using a DerSimonian-Laird random-effects model, according to study design. Risk of bias assessment and sensitivity analyses were conducted. 44 eligible studies were included, which consisted of 58,468,404 subjects from 16 countries. Compared with non-DM patients, DM patients had 3.59–fold (95% confidence interval (CI) 2.25–5.73), 1.55–fold (95% CI 1.39–1.72), and 2.09–fold (95% CI 1.71–2.55) increased risk of active TB in four prospective, 16 retrospective, and 17 case-control studies, respectively. Country income level (3.16–fold in low/middle–vs. 1.73–fold in high–income countries), background TB incidence (2.05–fold in countries with >50 vs. 1.89–fold in countries with ≤50 TB cases per 100,000 person-year), and geographical region (2.44–fold in Asia vs. 1.71–fold in Europe and 1.73–fold in USA/Canada) affected appreciably the estimated association, but potential risk of bias, type of population (general versus clinical), and potential for duplicate data, did not. Microbiological ascertainment for TB (3.03–fold) and/or blood testing for DM (3.10–fold), as well as uncontrolled DM (3.30–fold), resulted in stronger estimated association. DM is associated with a two- to four-fold increased risk of active TB. The association was stronger when ascertainment was based on biological testing rather than medical records or self-report. The burgeoning DM epidemic could impact upon the achievements of the WHO “*End TB Strategy”* for reducing TB incidence.

## Introduction

Despite the decline in the mortality rate of active tuberculosis (TB) since 1990, TB is ranked as one of the leading causes of death [1]. In 2015, there were an estimated 10.4 million incident TB cases worldwide [1]. The “*End TB Strategy*” launched by the World Health Organization (WHO) in 2016, aims to end the global TB epidemic by 2035 [1]. Targets set in this strategy include 90% reduction in TB deaths and an 80% reduction in TB incidence by 2030, compared with 2015 [1].

The growing epidemic of diabetes mellitus (DM) is set to become one of the major global health challenges [2]. The number of individuals with DM is projected to rise from 415 million in 2015 to 642 million by 2040 [3]. It is estimated that over a million TB cases among adults were affected by DM in 2012 [4]. The rising DM epidemic could contribute to an increase in TB burden. Although a few studies have failed to confirm a positive association between TB and DM [5–7], other studies reported a strong association between DM and active TB [8–12]. Based on earlier published summary effect estimates, DM increases the risk of active TB by 3.11–fold [13] and latent TB by 1.18–fold [14]. DM also has a major effect on TB treatment outcomes [15, 16]; in particular, it is associated with delayed sputum culture conversion, increased risk of treatment failure, and increased risk of TB relapse and mortality [17]. With the accumulation of recent evidence supporting the TB–DM association, there is a need for an updated understanding of the magnitude of the TB–DM association. This understanding is critical for implementation of comprehensive TB and DM control programs.

In this study, we aimed to systematically review the published literature on the association between active TB and DM, and to statistically summarize the evidence on the strength of the association.

## Materials and methods

### Search strategy and selection criteria

This review follows Cochrane Collaboration guidelines [18] and reports findings using the Preferred Reporting Items for Systematic reviews and Meta-analyses (PRISMA) guidelines [19].

We searched Medline (from 1945 to December 22, 2015) and EMBASE (from 1980 to December 22, 2015) databases, for studies on the TB–DM association. For inclusiveness, our search strategy covered studies that examined any risk factor for TB. The literature search protocol is summarized in the S1 Box.

Inclusion of studies was restricted to human studies that provided an estimate or allowed us to compute an estimate of the association, adjusted at least for one variable. No restrictions were made on study language, population, publication year, or region.

We excluded studies: amongst animals or children, if qualitative in design, case reports, case series, reviews, anonymous reports, editorials or author commentaries, with no appropriate control arm or comparator group, where TB patients with DM were not separated from those with other co-morbidities, of TB outcomes rather than the association, that did not report adjusted estimates of the TB–DM association, and duplicate reports.

**Fig 1:**
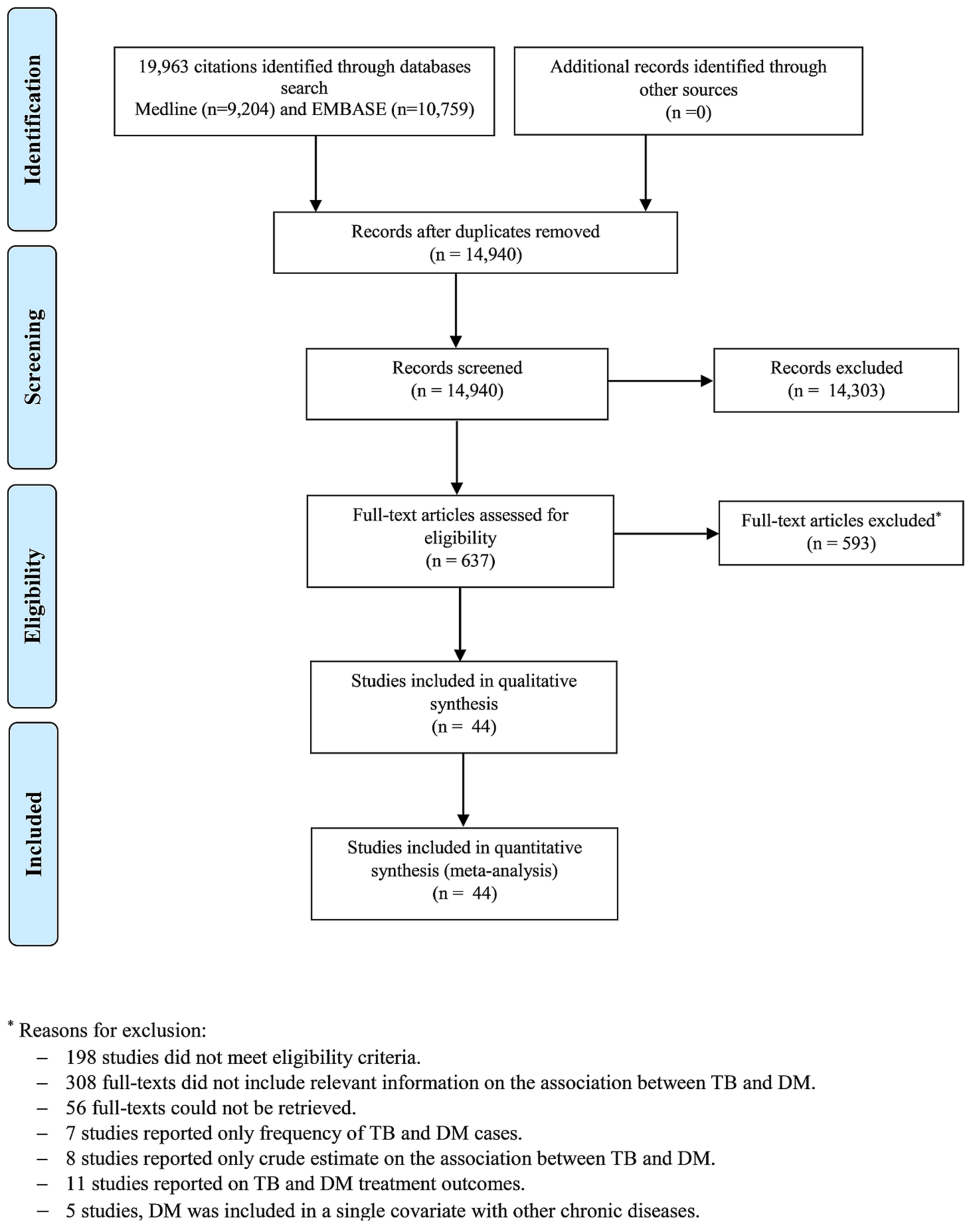
Flow diagram of study selection. Published studies were retrieved from the MEDLINE-PubMed and EMBASE databases. TB: tuberculosis; DM: diabetes mellitus.

We contacted authors of potentially eligible studies to provide the adjusted estimate for the association, if the adjusted estimate was not included in the publication. Studies whose authors did not respond were excluded. The flow diagram of study selection is shown in Fig 1. First author (RHA) screened all titles and abstracts, reviewed full-text articles, and assessed their eligibility for inclusion.

### Data extraction and quality assessment

Three authors (RHA, JAC, and LJA) designed the literature search terms and strategy. All potentially relevant full-text articles retrieved and reviewed to confirm eligibility. If eligible, data were extracted. All authors (RHA, FP, JAC, and LJA) contributed in assessing the eligibility of the included studies. The first author (RHA) extracted the data, which were re-extracted independently by at least one co-author (FP, JAC, or LJA). Discrepancies in data extraction were resolved by consensus or consultation with a third co-author. In addition to extracting baseline characteristics of included studies, we assessed methodological aspects, such as sampling strategy, characteristics of the study population, and TB and DM ascertainment. If a study reported more than one adjusted estimate or stratified adjusted estimates, we chose the most representative and relevant estimate with the most confirmatory DM (i.e. prioritizing HbA1c over fasting blood glucose (FBG)) and/or TB (i.e. prioritizing bacterial culture over X-ray) ascertainment strategy, or the estimate adjusted for the largest number of appropriate variables when the study reported multiple adjustment models. Five of the contacted authors have provided us with adjusted estimates [7, 20–23]. Two adjusted estimates [24, 25] were extracted from a previous review [13].

We evaluated each study’s risk of bias (ROB) using nine domains for cohort and cross-sectional studies, and eleven domains for case-control studies. The ROB domains were adapted from Cochrane guidelines for systematic reviews [26, 27] and other validated quality assessment tools [28, 29]. The utilized ROB domains are presented in the S2 Box and were related to different quality criteria such as rigor of sampling strategy, TB and DM case definition and ascertainment, and DM timing in relation to TB. In case-control studies, convenience sampling of TB cases was considered as a probability-based sampling method as long as all cases in the sampling frame were selected. Based on the composite scores of the ROB domains, each cohort or cross-sectional study was classified as “potentially of low ROB” (score ≥7) or “potentially of high ROB” (score <7). Each case-control study was classified as “potentially of low ROB” (score ≥9) or “potentially of high ROB” (score <9) (S2 Box).

### Statistical analysis

For studies reporting stratified crude estimates, we calculated an overall adjusted estimate by only one stratification if there was an overlap with other strata. In such studies, we prioritized pooling crude estimates that stratified by co-morbidity, location, age, or sex, consecutively. When there were two or more levels of stratification without overlap, we pooled estimates for this sub-levels stratification. If a study stratified estimates according to DM type, we pooled the overall adjusted estimate regardless of the DM type.

In sensitivity analyses, we pooled estimates of studies of “potentially low ROB”, studies unlikely to contain duplicate individual–level data, and studies in the general population rather than specific clinical populations. We further pooled estimates stratified by potential for duplicate data on same patients, country-income level, background TB incidence (≤50 or >50 cases per 100,000 person-year), region, and TB and DM ascertainment methodology. We obtained relevant data on TB incidence from the included studies or from the closest matching year made available by public databases.

We pooled adjusted estimates stratified by study design and regardless of study design using random-effects model [30]. Cochran’s Q statistic was used to test for evidence of heterogeneity [31, 32]. We estimated the I-squared (*I*^*2*^) as a measure of heterogeneity. We computed Tau-squared (*τ*^2^) to estimate the between-study variance of the true association between TB and DM [31, 32].

We assessed the presence of publication bias by examining the funnel plots using Egger’s *t* statistic to examine asymmetry (S2 Fig) [33]. We used the pooled effect estimate in four prospective studies to estimate the attributable risk fraction of DM in developing active TB among people with DM and to estimate the population attributable risk fraction of DM in developing active TB among the entire population in six high-TB-burden countries (India, Indonesia, China, Nigeria, Pakistan, and South Africa), that accounted for 60% of new TB cases in 2015 [1]. Calculations are presented in the S1 Text.

All Statistical analyses were performed using STATA SE 14 (Stata Corporation, College Station, TX) [34].

## Results

We identified 19,963 publications, 44 of which were found relevant and included in this systematic review and meta-analysis (Fig 1) [5–12, 20–25, 35–64]. The included studies consisted of 58,468,404 subjects and 89,592 TB cases and they were set in 16 countries. Most studies were conducted in Taiwan (11 studies) and USA (11 studies), while only one study was in Africa [59]. Two studies of different designs stratified patients according to DM type (1 or 2) [9, 51], one study was in patients with type 1 DM [48], while the rest of the studies were either among type 2 DM patients or the type of DM was not specified (presumably, type 2 DM as it is more prevalent). There were four prospective [8, 23, 35, 36], 19 retrospective [5–7, 20, 22, 24, 37–49], 17 case-control [9–11, 21, 25, 50–61], and three cross-sectional [12, 62, 63] studies. One study was classified as “other” as the exact study design could not fit into the other categories [64]. One of the prospective studies was among people aged ≥65 years [35] and one was among renal allograft recipients [36]. Four of the retrospective studies were among renal patients [7, 24, 37, 41]. Several studies were national in scope, thereby including the national population as the study sample size, such as for a study from Australia [47]. Seven retrospective studies in Taiwan [22, 37, 40–43, 48] and one retrospective and one case-control-study in the United kingdom, and three cross-sectional [12, 62, 63] studies. One study was classified as “other” as the exact study design could not fit into the other categories [64]. One of the prospective studies was among people aged ≥65 years [35] and one was among renal allograft recipients [36]. Four of the retrospective studies were among renal patients [7, 24, 37, 41]. Several studies were national in scope, thereby including the national population as the study sample size, such as for a study from Australia [47]. Seven retrospective studies in Taiwan [22, 37, 40–43, 48] were potentially duplicate studies using the same database with overlapping years. One cross-sectional study was set in 46 countries and one retrospective and one case-control-study in the United kingdom [38, 53] were potentially duplicate studies using the same database with overlapping years. One cross-sectional study was set in 46 countries [62].

In prospective studies, estimates were adjusted at least for age except for one study that reported sex-specific crude estimates [8]. For the latter we pooled these for the present review. In one prospective study the effect estimate was not adjusted for sex [36]. In retrospective studies, all estimates were adjusted at least for age or sex. In case-control studies, estimates were adjusted at least for age and sex except in two studies where the estimate was adjusted for age but not for sex [54, 55]. All cross-sectional studies were adjusted at least for age and sex. Baseline characteristics of all included studies are shown in Tables 1 and 2.

**Table 1:** Baseline characteristics of 23 cohort studies, prospective and retrospective, that reported on the association between TB and DM and that were included in the meta-analyses.

First author, year	Country	Study period	Study location	Study population	DM ascertainment	TB ascertainment	Sample size	TB cases	Adjusted effect size (95% CI)	TB incidence/100,000 p–y^1^	Adjusted variables
**Prospective**											
Kim et al (1995) [8]	South Korea	1988–1990	Authorized hospitals in South Korea	Civil servants examined by Korean Medical Insurance Corporation who claimed health insurance for TB	DM ascertained by glucose level of ≥119 mg/dl at screening followed by FBG ≥150 mg/dl & PPBG ≥180 mg/dl	Pulmonary TB, bacteriologically ascertained	814,713	5,105	RRs: 4.97 (3.68–6.70)	306	Sex-specific stratum crude RRs were pooled using random-effects model^2^
Leung et al (2008) [35]	China	January, 2000–December, 2005	18 elderly health service centers	Elderly people aged ≥65 years	DM ascertained by HbA1c ≥7% at enrollment for those with known history of DM based on a physician diagnosis	Culture confirmed pulmonary and extra-pulmonary TB	42,116	326	HR: 2.69 (1.94–3.72)	90	Age, sex, alcohol use, BMI, marital status, smoking, language, education, housing, working status, public means-tested financial assistance status, CVD, hypertension, COPD/asthma, malignancy, recent weight loss of 5% within 6 months, hospital admission within 12 months, & activities of daily living scores
						Active TB			HR: 2.56 (1.95–3.35)		
						Pulmonary TB			HR: 2.80 (2.11–3.70)		
						Extrapulmonary TB			HR: 0.88 (0.35–2.20)		
John et al (2001) [36]	India	1986–1999	Christian Medical College and Hospital at Vellore in southern India	Renal allograft recipients	DM ascertained by FBG >120 mg/dl, or 2-hours PPBG 200>mg/dl, or two elevated levels of either measurement from medical records	All TB ascertained from medical records based on X-ray, AFB, gastric juice, bronchoalveolar specimen, or histopathology	1,251	166	HR: 2.24 (1.38–3.65)	168^3^	Age, chronic liver disease, deep mycoses, cytomegalovirus, *Pneumocystis carinii* pneumonia, nocardia, prednisolone plus azathioprine, & cyclosporine use
Cegielski et al (2012) [23]	USA	1971–1992	General population	Civilian, non-institutionalized adults aged 25–74 years recorded in the First National Health and Nutrition Examination Survey (NHANES I)	DM ascertained by self-report. NHANES I questionnaire asked respondent *'Has a doctor ever told you that you have any of the following conditions*, *and if so*, *do you still have it*? *Diabetes*? to assess DM status	All TB. 21 TB cases were ascertained by self-report, the rest based on the ICD-9–010–018 and 137, excluding TB exposure without disease (ICD-9-V01.1), primary infection without disease (ICD-9-010.0), TST positivity without diseases (ICD-9-795.5) & subjects who had TB before NHANES I	14,189	61	HR: 7.58 (2.94–19.49)	15.4^3^	Age, sex, & BMI
**Retrospective**											
Chung et al (2014) [22]	Taiwan	1997–2010	General population	Newly diagnosed TB patients from the Taiwan’s National Health Insurance Research Database and non-TB subjects from general population	DM ascertained by ICD-9-CM 250 codes from medical records	All TB ascertained by receiving medical care at least three times, including out-patient visits and/or hospitalizations, for a principal diagnosis of TB based on ICD–9–CM 011–018 codes	50,840	10,168	RRs: 1.55 (1.47–1.64)	72.5	Age & sex^5^
Ou et al (2012) [37]	Taiwan	January, 1997–December, 2006	General population	Kidney transplant recipients identified from the Taiwan’s National Health Insurance Database	DM ascertained from National Health Insurance Database	Newly diagnosed all TB ascertained by ICD–010–018 codes validated by the use of at least 2 anti-TB medications	4,554	109	OR: 1.42 (0.96–2.09)	67.4	Age, sex, COPD, autoimmune disease, cirrhosis, hepatitis C virus infection, HIV, cyclosporine, & mycophenolate mofetil
Chen et al (2013) [5]	China	2006–2008	General population in rural areas	Residents of Danyang county of Jiangsu province and Xiangtan county of Hunan province	DM ascertained by self-reported history of DM by answering the question ‘‘*Has a doctor ever told you that you have diabetes*?”	All TB ascertained by sputum smear positive (including scanty positive) or sputum culture-positive for *mycobacterium tuberculosis*	177,529	117	RRs: 2.43 (0.84–7.00)	59.74 in Danyang county. 101.1 in Xiangtan county	County-specific aRRs for sex, age, marital status, occupation, & educational level were pooled using random-effects model^2^
Pealing et al (2015) [38]	United Kingdom	1990–2013	Clinical practice research data linked to the hospital episode statistics	DM cohort: patients with first recorded diagnosis for DM (type 1 and 2) aged ≥5 years.DM-free cohort: patients who did not have a prevalent diagnosis of DM on the matched index date	DM ascertained by HbA1c >7.5% mmol/mol	All TB ascertained by ICD–10 codes. Prescriptions for anti-TB drugs were not used in developing or later validating cases of TB identified by diagnostic codes. Only one TB case occurred in T1DM cases	6,941,000	969	RR: 1.30 (1.01–1.66)	13.9 in 2012	Age, smoking, alcohol use, BMI, ethnicity, & index of multiple deprivations. DM & non-DM subjects matched by age ±5yeras, sex, & general practice
Moran-Mendoza et al (2010) [6]	Canada	1990–2000	General population in British Columbia	Contacts of active TB cases recorded at the Division of TB control at the British Columbia Center for Diseases Control, excluding contacts of HIV infection cases or previous active TB cases	DM ascertained from databases, but unclear which databases	All TB by smear and/or culture positive for tubercle bacilli, histopathological diagnosis, or clinical and radiological diagnosis of active TB, with complete treatment response, when smears and cultures were negative	33,146	228	HR: 1.76 (0.54–5.75)	7^2^	Age, sex, malignancy, corticosteroids, alcohol, malnutrition, closeness of TB contact, TST size in millimeter, intravenous drug use, ethnicity, SES, recent arrival from country with high TB prevalence, residents/employees in prisons, nursing homes or homeless shelters, chest X-ray compatible with previous TB, & previous BCG vaccination. Adjustment was done with robust variance estimation
Baker et al (2012) [39]	Taiwan	August, 2001–December, 2004	General population	Taiwanese adults aged ≥12 years interviewed during the Taiwan’s 2001 National Health Interview Survey (NHIS)	Treated DM ascertained by ≥2 outpatient ICD–9–CM codes for DM, ≥1 inpatient ICD–9–CM code for DM, or prescription of anti-DM medications for ≥28 days during the study period or ≥2 prescriptions	All TB ascertained if all of the following criteria reported in NHIS database: ≥1 medical visit during the follow-up period with an ICD–9–CM code for TB (codes 010–018); a prescription for ≥2 anti-TB medications for >28 days during the study period; and no finding of a misdiagnosis of TB during the study period on the basis of later diagnosis of non-TB mycobacterial infection, lung cancer, or TB infection without evidence of disease	17,715	57	HR: 2.60 (1.34–5.03)	73	Age, sex, income, employment, alcohol use, education, BMI, living in a crowded home, receipt of government subsidy, residence in an indigenous community, hypertension, heart disease, & lung disease
Kuo et al (2013) [40]	Taiwan	2000–2011	General population	Patients aged ≥18–≥70 years with type 2 DM matched by sex, year of birth, and month and year of first diagnosis at enrollment with patients without DM or TB recorded in the Taiwan’s National Health Insurance Research Database representing about 5% of Taiwan’s population, excluding HIV cases	DM ascertained by ICD–9–250 (excluding 2501) with continuous prescriptions of anti-DM medications for ≥60 days	All TB ascertained by ICD–9–010–018 codes with continuous prescriptions of anti-TB medications for ≥60 days at least one year after DM code	253,349	5,013	HR: 1.31 (1.23–1.39)	73 [65]	Age, sex, bronchiectasis, asthma, & COPD
Hu et al (2014) [41]	Taiwan	January, 1998–December, 2009	General population	Patients receiving dialysis recorded in the Taiwan’s National Health Insurance Research Database, representing about 5% of Taiwan’s population in 2000	DM ascertained by ICD–9–250 or A181	All TB ascertained by ICD–9–010–018 or A02 codes & ≥2 anti-TB medications for >28 days	20,655	287	HR: 1.36 (1.05–1.76)	64.89 in 1998.	Age, sex, hypertension, silicosis, COPD, connective tissue diseases, & malignancy co-morbidities
										75 in 2002	
										67 in 2003	
										74 in 2004	
										72.5 in 2005	
										67 in 2006	
										63 in 2007	
										62 in 2008 [65]^4^	
Lee et al (2013) [42]	Taiwan	1996–2007	General population	Subjects with and without COPD disease matched in age (within 5 years), sex, and time of entering the Longitudinal Health Insurance Database-2005 recorded in the National Health Insurance program database that covers more than 95% residents of Taiwan since 1996	DM ascertained from Longitudinal Health Insurance Database–2005	All TB ascertained by at least two ambulatory visits or one inpatient record with a compatible diagnosis (ICD–9–CM codes 010–012, and 018, and A-codes A020, A021), plus at least one prescription consisting of ≥3 anti-TB. There should be a prescription of at least 2 anti-TB drugs simultaneously for ≥ 120 days during a period of 180 days	23,594	674	HR: 1.25 (1.02–1.53)	64.89 in 1998	Age, sex, oral corticosteroids, inhaled corticosteroids & oral β-agonists
										75 in 2002	
										67 in 2003	
										74 in 2004	
										72.5 in 2005	
										67 in 2006	
										63 in 2007 [65] ^4^	
Wu et al (2011) [43]	Taiwan	January, 2000–December, 2007	General population	New onset cancer patients and cancer-free patients recorded in the Taiwan’s’ National Health Insurance Database in 2005, matched by sex and age	DM ascertained by ICD–9–250	All TB ascertained by ICD-9-010–018 with prescription history of treatment with isoniazid	82,435	694	HR: 1.38 (1.17–1.62)	64.89 in 1998	Age, sex, chronic renal failure, autoimmune diseases, COPD, aerodigestive tract and lung cancers, haematological cancers, & other major/less common cancers
										75 in 2002	
										67 in 2003	
										74 in 2004	
										72.5 in 2005	
										67 in 2006	
										63 in 2007 [65] ^4^	
Chen et al (2006) [24]	Taiwan	January, 1983–December, 2003	Hospitals	Renal transplant recipients in Taichung	DM ascertained from medical records	All TB ascertained either by positive culture, presence of caseating or non–caseating granuloma in biopsy specimens taken from involved tissue and responsive to treatment, or typical chest X–ray finding or clinical presentation consistent with TB, without microbiological or pathological confirmation but with favorable response to anti-TB treatment	756	29	RRs: 3.07 (1.14–8.26)	66.67	Age, sex, dialysis duration, hepatitis B virus infection, hepatitis C virus infection, graft rejection >3, & immunosuppressive medications. Adjusted effect estimate reported in the previous review [13]
Rungruanghiranya et al (2008) [7]	Thailand	January, 1992–December, 2007	Nationwide	Renal transplant recipients	DM ascertained from case medical records	All TB ascertained by one or more of: AFB in body fluid smears, TB-polymerase chain reaction, and/or growth in various culture specimens; histopathology examination of tissue specimens showing either AFB or granulomatous inflammation; response to TB treatment in patients with typical radiographic findings consistent with TB, or those who had fever of unknown origin despite negative results of extensive investigations	233	9	OR: 3.59 (0.74–17.35)	142 in 2005	Age & sex^5^
Demlow et al (2015) [44]	USA	2010–2012	California department of public health	Non-institutionalized TB cases with and without DM aged ≥18 years	DM ascertained based on history of DM gathered from medical records or healthcare provider, excluding pre-DM, borderline DM, self-reported DM, or gestational DM	All TB ascertained based on information gathered from local TB control programs from medical records or a health care provider	27,797,000	6,050	RRs: 2.18 (1.79–2.66)	4.8	Age & birth location-specific stratified crude RRs were pooled using random-effects model^2^
Suwanpimolkul et al (2014) [45]	USA	April, 2005–March, 2012	San Francisco TB control sections	All individuals seeking medical care who had final diagnosis of TB, latent TB (LTB), or no evidence of TB or LTB. DM in TB patients was assessed versus DM in individuals with LTB	DM status reported by patient ascertained from medical records based on the screening policies of the San Francisco TB control sections	All TB ascertained by Standards of the American Thoracic Society and Centers for Disease Control and Prevention	5,162	791	OR: 1.81 (1.37–2.39)	2.8	Age & place of birth
Kamper-Jorgensen et al (2015) [20]	Denmark	January, 1995–December, 2009	General population	Entire Danish population	DM ascertained from Danish National Diabetes Register including blood glucose testing, foot treatment, or purchase of anti-DM drugs	All TB ascertained according to the WHO definitions. TB is diagnosed on the basis of microbiology and/or laboratory results, or solely on clinical evaluation. In Denmark, around 70–75% of all notified cases are verified using culture	77,935	6,468	RR: 1.60 (1.43–1.79)	7^3^	Age & sex^5^
Young et al (2012) [46]	England	ORLS1: 1963–1998.ORLS2: 1999–2005	Admissions records in all NHS hospitals in defined populations in the former Oxford NHS region	DM cohort: all forms of DM first record on file for each individual with DM. Reference cohort: people with various common orthopedic, dental, ENT and other relatively minor disorders	DM ascertained by ICD7 260, ICD8 250, ICD9 250, ICD10 E10-E14 codes	All TB ascertained by ICD7 001–019, ICD8 010–019, ICD9 010–018, 137, ICD10 A15–A19, B90 codes	837,399	7,996	RR: 2.02 (1.35–3.04)	56 in 1964	Age in 5–years band, sex, time period, & district of residence adjusted ORLS1 & ORLS2 survey rounds-specific RRs were pooled using random-effects model^2^
										26 in 1974	
										13 in 1984	
										4 in 1994	
										5 in 2004 [65] ^4^	
Dobler et al (2012) [47]	Australia	January, 2001–December, 2006	General population	Residents of Australia	DM ascertained from medical records per the National Diabetes Services Scheme	Culture–positive TB ascertained based on state and territory TB notification records.	19,855,283	6,276	RRs: 1.49 (1.05–2.11)	5.8	Age, sex, indigenous status, & TB incidence in country of birth
						All TB			RRs: 1.48 (1.04–2.10)		
						All TB in insulin users			RRs: 2.27 (1.41–3.66)		
						Culture–positive TB in insulin users			RRs: 2.55 (1.62–4.01)		
Shen et al (2014) [48]	Taiwan	2002–2011	General population	Type 1 DM patients aged <40 years identified from the Registry of Catastrophic Illnesses Patient database & non-type 1 DM cohort identified from the Longitudinal Health Insurance Database in 2000	Newly diagnosed type 1 DM ascertained by ICD–9 250.x1 & 250.x3 codes from data recorded in the Registry of Catastrophic Illnesses Patient database	All TB ascertained by ICD–9–CM codes from medical records	25,975	59	HR: 4.23 (2.43–7.36)	53	Age, sex, chronic liver infection, chronic kidney infection, & previous infections
Dyck et al (2007) [49]	Canada	January 1986–December 2001 for TB case; January 1991–December 1995 for DM survey	General population	Registered American Indians and other Saskatchewans aged ≥20 years selected from population-based health databases in Saskatchewan	DM ascertained by ICD–9: 250 codes from medical charts	All TB cases aged ≥20 years reported to Saskatchewan Health	791,673	1,118	RR: 1.00 (0.69–1.44)	43.8	Age, race, & sex stratum-specific crude ORs were pooled using random–effects model^2^
**Other**^6^											
Ponce-De-Leon (2004) [64]	Mexico, state of Veracruz	March 1995–April 2003 for TB case; 2005 for DM survey	General population	Non–institutionalized civilians	DM ascertained by a previous diagnosis of a physician; or FBG ≥126 mg/dl or random blood glucose ≥200 mg/dl	All TB ascertained by positive AFB or positive culture	21,230	581	RR: 6.00 (5.00–7.20)	28	Age & sex–standardized for the adult population of the study area

^1^ Background TB incidence per 100,000 person–year during the same year or closest year to the survey.

^2^ Pooling was done by the present study team and was not reported in the original study.

^3^ Data obtained from external source; the World Bank records (http://www.cdc.gov/tb/statistics/tbcases.htm) and the WHO TB country profiles (http://www.who.int/tb/country/data/profiles/en/)

^4^ Data retrieved from (http://www.cdc.gov.tw/uploads/files/201407/103228a0-fadd-47b0-b056-8dedda9fce1d.pdf); (file:///C:/Users/rha2006/Downloads/%253f44CurrentStatusofTuberculosisinTaiwan%20(1).pdf).

^5^ Adjusted estimate provided by author.

^6^ Study by Ponce-De-Leon A., et al [64] neither categorized as prospective, retrospective, cross–sectional, or case–control study.

TB: tuberculosis; DM: diabetes mellitus; HbA1c: glycated haemoglobin (measure of serum glucose levels over time in humans); PPBG: postprandial blood glucose; AFB: acid–fast bacilli; COPD: chronic obstructive pulmonary disease; TST: tuberculin skin test; HIV: human–immunodeficiency virus; RRs: relative risk; OR: odds ratio; HR: hazard ratio; RR: rate ratio; aOR: adjusted odds ratio; aRRs: adjusted relative risk; BMI: body mass index; BCG: bacilli Calmette–Guérin; ICD–9: International Statistical Classification of Diseases and Related Health Problems 9th edition; WHO: World Health Organization; CDC 1990: 1990 Case Definition for Tuberculosis by Center for Disease Control (US).

**Table 2:** Baseline characteristics of 17 case–control and 3 cross–sectional studies that reported on the association between TB and DM and that were included in the meta-analyses.

First author, year	Country	Study period	Study location	Study population	DM ascertainment	TB ascertainment	TB cases	Controls	Adjusted effect size OR (95% CI)	TB incidence/ 100,000 p–y^1^	Matched/Adjusted variables
**Case-control**											
Alisjahbana et al (2006) [10]	Indonesia	March, 2001– March, 2005	Central Jakarta	Cases: TB-patients aged >15 years from outpatients TB-clinics. Controls: TB-free individuals from TB cases communities	DM ascertained by FBG ≥126 mg/dl after stopping taking anti-diabetic agents for 48 hours & FBG were considered impaired for >110 and <126 mg/dl, in accordance with WHO criteria	Pulmonary TB ascertained by clinical presentation & chest X–ray examination confirmed by microscopic detection of AFB	454	556	4.70 (2.70–8.10)	128	Matched by: sex, age (±10%), & residential location. Adjusted for: age, sex, BMI, income, number of individuals per household, & presence of TB contact in family or household
Lai et al (2014) [50]	Taiwan	1998–2011	General population	Cases: newly diagnosed TB-patients aged ≥20 years selected from the National Health Insurance Program database. Controls: TB-free individuals from same database	DM ascertained by ICD–9 codes from medical records	Pulmonary TB ascertained by ICD–9–010, 011, 012, 018 codes from medical records	11,366	45,464	1.46 (1.38–1.54)	64.89 in 1998	Matched by: age, sex, & index year of TB diagnosis. Adjusted for: age, COPD, pneumoconiosi, chronic kidney disease, & chronic liver disease
										75 in 2002	
										67 in 2003	
										74 in 2004	
										72.5 in 2005	
										67 in 2006	
										63 in 2007	
										62 in 2008	
										89 in 2010	
										54.4 in 2011[65]	
Ku et al (2013) [21]	South Korea	1985–2012	Severance hospital, Ajou University hospital, & Wonju Christian hospital & Seoul medical center	Cases: HIV-1-infected TB-patients aged ≥18 years. Controls: HIV–1–infected TB–free individuals	DM ascertained from patient’s medical records	All TB ascertained by isolation of *Mycobacterium tuberculosis* or demonstration of AFB from a clinical specimen or in a histopathological lesion when culture was not available in a patient with signs or symptoms compatible with TB, or evidence of resolution of disease where treatment with two or more anti-TB medications had been prescribed and follow-up had been instigated, excluding AFB TB–positive patients who were finally diagnosed with non-TB mycobacterial infection	170	340	1.53 (0.74–3.14)	119.3	Matched by: HIV status, & CD4+ T–cell count at first visit & the date of first visit. Adjusted for: age & sex^3^
Leegaard et al (2011) [51]	Denmark	January, 1980–December, 2008	Northern Danish population	Cases: first time hospital contact with principal diagnosis of TB obtained from DNRP who lived in Northern Denmark for ≥6 months since the TB diagnosis date. Controls: TB-free individuals from Danish civilian registration system	DM ascertained by in- or outpatient hospital contact involving diabetes, any use of oral anti-diabetes drugs or insulin, at least one visit to a chiropodist for diabetes foot care, at least five glucose-related services in general practice in 1 year, or at least two glucose-related services each year during 5 subsequent years. Patients under 30 on therapy = Type 1DM, rest Type 2DM. HbA1c where available for a subset of controls and cases	All TB ascertained by ICD–8: 010–019; ICD–10: A15–A19 codes. A subset microbiologically confirmed TB	2,950	14,274	1.18 (0.96–1.45)	7	Matched by: age (±5 years), sex, country of origin, & place & length of residence in Denmark (±1 year). Adjusted for: age, sex, country of origin, place & length of residence in Denmark, comorbidities (myocardial infarction, congestive heart failure, peripheral vascular disease, CVD, dementia, COPD, connective tissue disease, ulcer disease, mild liver disease, hemiplegia, moderate to severe renal disease, any tumor, leukemia, lymphoma, metastatic solid tumor, & HIV/AIDS), alcoholism-related disorders, marital status, number of children <15 years, & degree of urbanization
					Type 1 DM				2.59 (0.44–15.29)		
					Type 2 DM				1.17 (0.95–1.44)		
Jurcev-Savicevic et al (2013) [52]	Croatia	2006–2008	Seven Croatian counties	Cases: TB-patients aged ≥15 years. Controls: TB-free individuals from database of general practitioners in each TB-case county who had not developed TB in a two-month period	DM ascertainment by self-report	Pulmonary TB ascertained by bacterial positive culture among cases	300	300	2.38 (1.05–5.38)	23	Matched by: age (±2 years), sex, & county of residence that had no history of TB from the database of general practitioners. Adjusted for: age, sex, BMI, country of birth of parents, education, household equipment, employment, smoking status, contact with TB, & malignant disease
Jick et al (2006) [53]	UK	1990–2001	General population	Cases: first–time TB–patients obtained from General Practice Research Database. Controls: TB–free individuals from same database	DM ascertained by presence of anti-DM medication prior to TB index date	All TB ascertained by prescription of at least 3 anti-TB medications for at least 6 months	497	1,966	3.80 (2.30–6.10)	3	Matched by: age, sex, geography, index date, & medical history. Adjusted for: age, sex, index date, amount of computerized medical history, glucocorticoid use, smoking, BMI, pulmonary disease, & use of anti-rheumatic / immunosuppressive agents
Pablos-Mendez et al (1997) [9]	USA	1991	Civilian hospitals in California	Cases: TB–patients. Controls: TB–free patients with primary discharge diagnosis of deep venous thrombosis of the legs, pulmonary embolism, or acute appendicitis	DM ascertained from medical charts coded as ICD–9 250.0–250.9	All TB coded as ICD–9 010 to 018	5,290	37,366	1.53 (0.81–2.90)	17.3^2^	Matched by: age & race. Adjusted for: race-specific aORs for age, sex, poor education, median income, health insurance, HIV-related conditions, chronic renal insufficiency, alcohol-related conditions, & drug use were pooled using random-effects model^5^
					Type 1 DM				1.40 (0.83–2.35)		
					Type 2 DM				1.02 (0.63–1.66)		
Perez et al (2006) [11]	USA	1999–2001	15 Texas/Mexico border counties	Cases: TB-patients aged ≥15 years from Texas hospitals discharge database. Controls: TB-free patients aged ≥15 years with deep venous thrombosis, pulmonary embolism, or acute appendicitis from same database. Excluding HIV cases	DM ascertained from medical chart coded as ICD–9: 250.0–250.9	All TB coded as ICD–9 code 010–018. TB codes were sought in the admitting diagnosis, principal diagnosis, and eight other variables with diagnosis codes	3,847	66,714	1.75 (1.32–2.33)	Mexico-borders counties: 13.1. Non-Mexico borders counties: 6.6	Region-specific aORs for age, sex, race/ethnicity, insurance type/status, any type of cancer, chronic renal failure, nutrition deficit, income, & education were pooled using random–effects model^5^
Corris et al (2012) [54]	USA	1976–1980	General population	Cases: TB–patients aged 20–74 years. Controls: TB–free individuals aged 20–74 years. Cases & controls were a cross-sectional sample from the second National Health and Nutrition Examination Survey included weighted civilian non–institutionalized US population	DM was ascertained by self-report to questions “*Do you have diabetes or sugar diabetes*?, "*Have you ever been told by a doctor that you have borderline diabetes*?" and/or a 75g OGTT test at current WHO cut points (OGTT takes precedence)	All TB ascertained by self-reported ever received diagnosis of TB from a doctor regardless of whether they still had it	166	15,191	2.31 (1.36–3.93)	11 in 1982^4^	Adjusted for: age, race, poverty index, BMI, household contact with TB, & cigarette smoking status
Buskin et al (1994) [55]	USA	1988–1990	Seattle/King county TB clinic	Cases: TB–patients, residents of King County aged >17 years seeking care at a TB clinic in Washington. Controls: active TB–free, residents of King County aged >17 years seeking care at a TB clinic in Washington	DM ascertained by self-reported history of DM taken from the questionnaire 1.7 months after the date of TB onset	All TB ascertained by CDC, 1990 criteria that emphasize laboratory confirmation of *Mycobacterium tuberculosis* and other specified criteria [66]	151	545	1.70 (0.70–4.30)	9	Adjusted for: age
Brassard et al (2006) [25]	USA	September, 1998–December, 2003	General population	Cases: TB–infected rheumatoid arthritis patients aged ≥18 years. Controls: TB–free rheumatoid arthritis patients aged ≥18 years. Cases & controls selected from the PharMetrics database with ≥1 prescription for antirheumatic medication	DM ascertained from medical chart coded as ICD-9 250.0–250.9	All TB ascertained from medical chart using ICD–9 code 010–018 codes	386	38,600	1.50 (1.15–1.90)	5.6	Matched by: date of cohort entry. Adjusted for: age, sex, silicosis, chronic renal failure, hemodialysis, solid organ transplant, head & neck cancer, NSAIDs, steroids, Cox-2 inhibitors. Adjusted effect estimate reported in the previous review [13]
Mori et al (1992) [56]	USA	January, 1983–December, 1989	Shannon county, South Dakota	Cases: American Oglala Sioux Indian TB–patients >18 years. Controls: TB–free individuals with positive TST before the median date of diagnosis of TB, August 1, 1986. Obtained from Oglala Sioux Indians from Indian health service hospital database	DM ascertained by anti-DM oral treatment (hypoglycemic agents or insulin); or ≥11.1 mmol/l at screening or ≥7.8 mmol/l FBG	Cases: clinically diagnosed TB from Indian health service and clinical charts based on the State Health Department definition of active TB. TB type not specified. Controls: positive TST ascertained from their medical records	46	46	5.20 (1.22–22.10)	90.9 in Shannon county	Matched by: age & residence. Adjusted for: sex, alcohol abuse, & isoniazid therapy for >6 months. Cases and controls were not significantly different according age
Viney et al (2015) [57]	Republic of Kiribati	June, 2010–March, 2012	Residents of South Tarawa city	Cases: TB–patients >18 years from the National TB Control Center and the National TB Laboratory. Controls: TB–free individuals >18 years (members of the same community without symptoms of TB)	DM ascertained by HbA1c ≥6.5% mmol/mol or self-reported DM with a treatment by a clinician	Cases: all TB ascertained by bacteriological, clinical and radiological criteria assessed by experienced physicians. Controls: all TB ascertained by TB-symptoms (cough >2 weeks, fever, nights sweats, weight loss), confirmed by TST	275	499	2.80 (2.00–4.10)	429	Adjusted for: age & sex
Coker et al (2006) [58]	Russia	January, 2003–December, 2003	Residents in the city of Samara	Cases: newly diagnosed adult TB–patients at any of city’s specialist TB clinics and recruited to a WHO DOT program. Controls: TB–free general residents of the Samara city	Method of DM ascertained was an unclear	Pulmonary TB ascertained by positive bacterial culture	334	334	7.83 (2.37–25.89)	118^2^	Matched by: age & sex. Adjusted for: age, sex, relative with TB, alcohol, drinking raw milk, assets, number of cohabitating person, employment, smoking, financial security, illicit drugs, & imprisonment
Faurholt-Jepsen et al (2011) [59]	Tanzania	April, 2006–January, 2009	Four major health facilities in Mwanza city	Cases: TB–patients aged ≥15 years, excluding pregnant or lactating women, patients terminally ill from TB or HIV, patients suffering from other severe diseases, & non–residents of Mwanza City. Controls: TB–free aged ≥15 years, with no history of TB in the household members and no evidence of active TB (cough, intermittent fevers, excessive night sweating in the past two weeks, and unexplained weight loss in the past month)	DM ascertained by either FBG >6mmol/L or OGTT >11mmol/L according to WHO guidelines, for both cases and controls	Pulmonary TB ascertained by initial diagnosis with sputum positive microscopy based on three sputum samples (‘‘spot-morning-spot”), with an additional early morning sputum sample was collected for *Mycobacterium tuberculosis* culture, for both cases and controls	803	350	2.13 (1.37–3.31)	504 in 2006	Matched by: residence, sex, age (± 5 years), not pregnant or lactating, not terminally ill from TB or HIV, not suffering from other diseases, & resident of Mwanaza city. Adjusted for: HIV–status stratum-specific aORs for age, sex, religion, marital status, & occupation were pooled using random–effects model^5^
										452 in 2009^2^	
Wu et al (2007) [60]	Taiwan	January, 2002–December, 2004	Chang Gung Memorial Hospital, Keelung	Cases: TB–patients with lower respiratory tract infection or who had been in contact with TB patients. Controls: non–TB pneumonia patients who did not meet the criteria for TB	DM ascertained from medical records	Pulmonary TB ascertained by positive sputum culture for *Mycobacterium tuberculosis*	264	438	3.43 (2.16–5.46)	75 in 2002	Adjusted for: age, sex, pneumoconiosis, bronchiectasis, liver cirrhosis, haemodialysis, & lung cancer
										67 in 2003	
										74 in 2004 [65]	
Rosenman and Hall, (1996) [61]	USA	January, 1985–May, 1987	New Jersey Department of Health	Cases: male TB-patients aged ≥35 years who speak English, excluding HIV positive and/or foreign born cases. Controls: TB-free individuals registered at the New Jersey Department of Health	DM ascertained by self-report	All TB ascertained by positive *mycobacterium tuberculosis* culture, or who had a physician diagnosis of pulmonary TB with multidrug anti-TB medication	148	290	1.16 (0.58–2.32)	9.5	Matched by: age (±5 years), gender, & race. Adjusted for: age, sex, & race
**Cross-sectional**											
Goldhaber-Fiebert et al (2011) [62]	Multi-center WHO survey	2002–2003	46 countries	General population	DM ascertained by self-report, based on positive response to the question "*Have you ever been diagnosed with diabetes (high blood glucose)*?"	All TB ascertained by self-reported symptoms of active TB, based on positive response to two questions "*Over the last 12 months*, *have you had blood in your phlegm or have you coughed blood*?" & "*Over the last 12 months*, *have you experienced cough lasting over 3 weeks*?"	124,545	1,744	1.81 (1.37–2.39)	-	Age, sex, BMI, schooling in years, smoking & length of being daily smoker, urban and rural residence, at least 1 drink per day, number of household members, number of individuals per room, & SES based on different household’s assets
Marks et al (2011) [12]	USA	2000–2005	General population	Civilians, non-institutionalized household residents aged ≥18 years selected from six national health insurance data bases	DM ascertained by self-report	All TB ascertained by self-report	190,350	668	1.40 (1.00–2.00)	7 in 2000	Age, sex, race/ethnicity, foreign birth, high school drop-out, history of homelessness or incarceration, ever cancer diagnosis, current cigarette smoking, past year alcohol abuse, no health insurance, & ever HIV testing
										5 in 2005^2^	
Wang et al (2013) [63]	China	September, 2010– December, 2012	TB clinics and neighboring communities in Linyi city	TB and non-TB patients with and without DM, excluding HIV positive patients	DM ascertained by FBG ≥7 mmol/L	Pulmonary TB ascertained by sputum smear positive; if sputum smears were negative and chest radiograph was compatible with active pulmonary TB, the patient was diagnosed as smear negative pulmonary TB	13,057	6,382	3.17 (1.14–8.84)	78 in 2010	Age, sex, BMI, family history of DM, annual income, education level, smoking, alcohol consumption, outdoor activity, & marital status
										73 in 2012^2^	

^1^ Background TB incidence per 100,000 person–year during the same year or closest year to the survey.

^2^ Data retrieved from (http://www.cdc.gov.tw/uploads/files/201407/103228a0-fadd-47b0-b056-8dedda9fce1d.pdf); (file:///C:/Users/rha2006/Downloads/%253f44CurrentStatusofTuberculosisinTaiwan%20(1).pdf).

^3^ Adjusted estimate provided by author.

^4^ Data obtained from external source; the World Bank records (http://data.worldbank.org/indicator/SH.TBS.INCD?end=2014&start=1990) and the WHO TB country profiles (http://www.who.int/tb/country/data/profiles/en/).

^5^ Pooling was done by the present study team and was not reported in the original study.

TB: tuberculosis; DM: diabetes mellitus; OR: odds ratio; aOR: adjusted odds ratio; HbA1c: glycated haemoglobin (measure of serum glucose levels over time in humans); DNRP: Danish National Registry of Patients; AFB: acid–fast bacilli; COPD: chronic obstructive pulmonary disease; TST: tuberculin skin test; SES: socio–economic status; HIV: human–immunodeficiency virus; BMI: body mass index; ICD–9: International Statistical Classification of Diseases and Related Health Problems 9th edition; WHO: World Health Organization; CDC 1990: 1990 Case Definition for Tuberculosis by Center for Disease Control (US); NSAID: non–steroidal anti–inflammatory drug; CVD: cardiovascular diseases.

The strongest estimate of the TB–DM association was 7.83 (95% CI 2.37–25.09) in a case-control study from Russia [58] followed by 7.58 (95% CI 2.94–19.49) in a prospective study from USA [23]. The lowest effect estimate was 1.00 (95% CI 0.69–1.44) in a retrospective study from Canada [49] followed by 1.16 (95% CI 0.58–2.32) in a case-control study from USA [61]. All four prospective studies demonstrated a positive association (*p*<0.05). Fourteen of the 19 retrospective [20, 22, 24, 38–48] and 12 of the 17 case-control studies [10, 11, 25, 50, 52–54, 56–60] demonstrated a positive association (*p*<0.05) between DM and TB (Tables 1 and 2).

**Fig 2:**
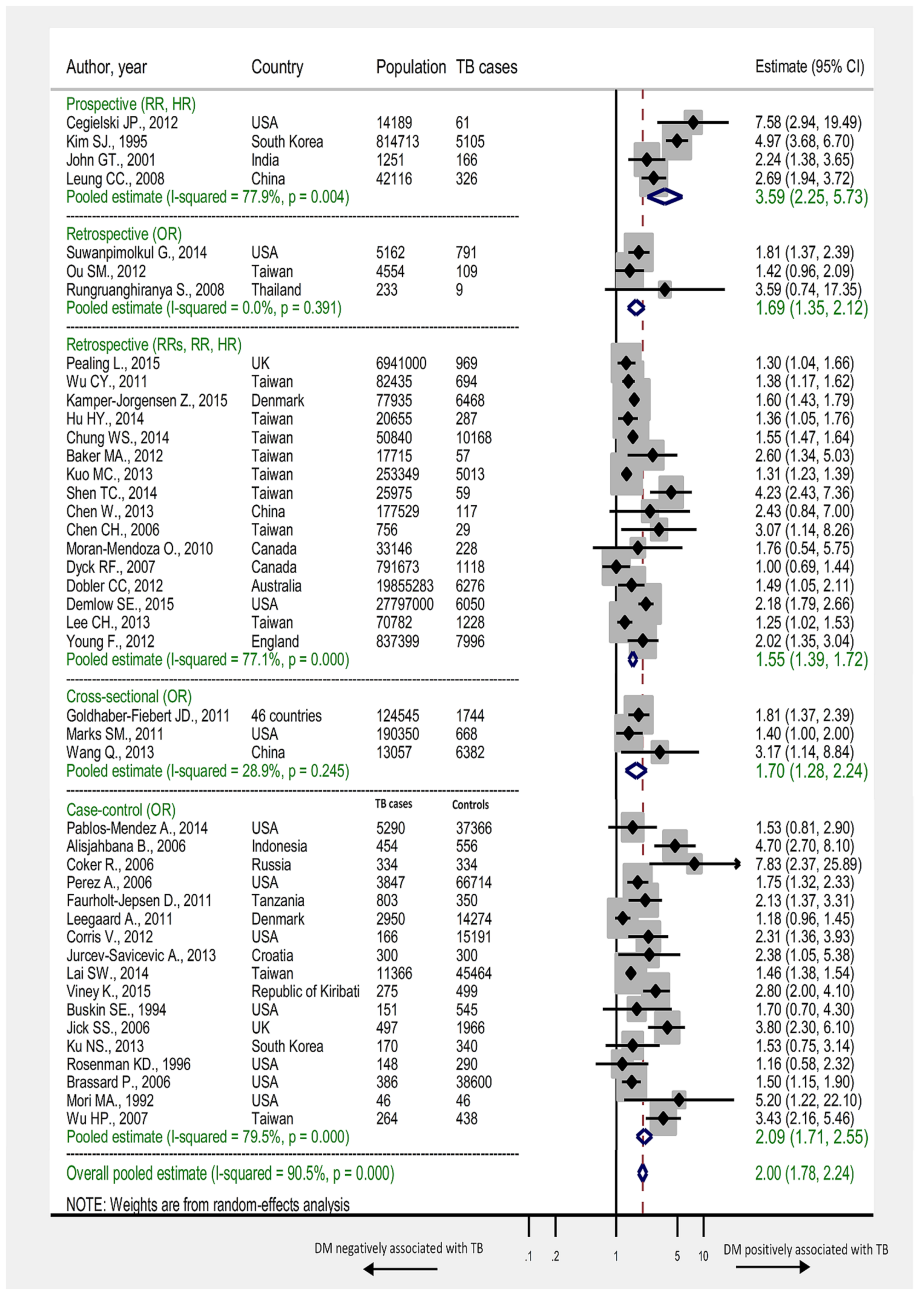
Forest plot of the meta-analyses. Pooled findings of 44 studies reporting adjusted estimates of the association between TB and DM, stratified according to study design. Size of the square is proportional to the precision (weight) of the study-specific effect estimates. Circle is the study–specific effect point estimate. Arrows indicate that the bars are truncated to fit the plot. The diamond is centered on the summary effect estimate, and the width indicates the corresponding 95% CI. RRs: relative risk; RR: rate ratio; OR: odds ratio; HR: hazard ratio.

In the four prospective studies, DM was associated with 3.59–fold (95% CI 2.25–5.73) increased risk of TB. The *I*^*2*^ was estimated at 77.9% indicating that most variation across studies was due to heterogeneity in effect size rather than chance. In 16 retrospective studies, DM was associated with 1.55–fold (95% CI 1.39–1.72) increased risk of TB (*I*^*2*^ = 77.1%). In the 17 case-control studies, DM was associated with 2.09–fold (95% CI 1.71–2.55) increased risk of TB (*I*^*2*^ = 79.5%). In the three cross-sectional studies, DM was associated with 1.70–fold (95% CI 1.28–2.24) increased risk of TB (*I*^*2*^ = 28.9%) (Table 3). In all studies regardless of study design, DM was associated with 2.00-fold (95% CI 1.78–2.24) increased risk of TB (*I*^*2*^ = 90.5%). Forest plots of meta-analysis according to study design are shown in Fig 2 with summary findings presented in Table 3.

**Table 3:** Summary findings of the meta-analyses for the association between DM and active TB, according to study design.

	Studies	Study population	Effect estimate		Pooled estimate		Heterogeneity measures		
	Total	Total	Measure of association	Range	Summary estimate	95% CI	*Q (p-value)*^1^	*τ*^*2*^ ^2^	*I*^*2*^ ^3^
**Study design**									
Prospective	4	872,269	RRs/HR	2.24–7.58	3.59	2.25–5.73	13.55 (*p* = 0.004)	0.1619	77.9%
Retrospective	16	56,990,255	RRs/RR/HR	1.00–4.23	1.55	1.39–1.72	65.45 (*p* < 0.0001)	0.0220	77.1%
Retrospective	3	9,949	OR	1.42–3.59	1.69	1.35–2.12	1.87 (*p* = 0.392)	0.0000	0.0%
Case-control	17	250,720	OR	1.16–7.83	2.09	1.71–2.55	77.88 (*p* < 0.0001)	0.1010	79.5%
Cross-sectional	3	327,952	OR	1.40–3.17	1.70	1.28–2.24	2.81 (*p* = 0.245)	0.0184	28.9%
Other^4^	1	21,230	RR	6.00	6.00	5.00–7.20	–^5^	–^5^	–^5^
**Overall**^6^	**44**	**58,472,375**	**OR/RRs/RR/HR**	**1.00–7.83**	**2.00**	**1.78–2.24**	**451.95 (*p* < 0.0001)**	**0.0945**	**90.5%**

^1^ Q: Cochran Q statistic is a measure assessing the existence of heterogeneity in estimates of association between TB and DM.

^2^ *τ*^*2*^: the estimated between–study variance in the true association between TB and DM estimates. The *τ*^*2*^ is for the variance of *beta* not for the back-transformed estimate.

^3^ *I*^2^: a measure assessing the magnitude of between-study variation that is due to differences in the association between TB and DM estimates across studies rather than chance.

^4^ Study by Ponce-de-Leon A., et al.,[64] neither categorized as prospective, retrospective, cross–sectional, or case–control study. Effect estimate is the individual study effect estimate.

^5^ Meta-analysis was not conducted due to limited number of studies (one study).

^6^ Overall estimate including risk ratios, rate ratios, hazard ratios, and odds ratios, that is regardless of the measure of association and study design. Background incidence rate of TB did not exceed 2 per 100 person-year in studies estimating an OR, therefore it is reasonable to assume that TB is sufficiently rare so that the ORs would estimate the risk ratios. Pooled estimate was implemented using a random-effects model.

RRs: relative risk; OR: odds ratio; HR: hazard ratio; RR: rate ratio; CI: confidence interval.

All of the four prospective studies were judged of “potentially low ROB”. Except one study [6], the 16 retrospective studies reporting relative risk (RRs), hazard ratio (HR), or rate ratio (RR), were judged of “potentially low ROB”. Of the 17 case-control studies, 13 were judged of “potentially low ROB” [9–11, 25, 50–54, 56, 57, 59, 61] (S1 Table).

In the sensitivity analyses presented in S1 Table, restricting the meta-analyses to studies judged of “potentially low ROB”, among only the general population, and with no potential for duplicate data on the same patients, DM patients were overall, that is by including all studies regardless of study design, at 2.00–fold (95% CI 1.77–2.27), 2.12–fold (95% CI 1.82–2.48), 1.63–fold (95% CI 1.45–1.82), respectively, increased risk of TB compared to the original overall estimate including all studies of 2.00–fold (95% CI 1.78–2.24) increased risk of TB (Table 3).

Moreover, overall, DM patients in low- or middle-income countries (3.16, 95% CI 2.20–4.53), in settings with TB incidence >50 cases per 100,000 person-year (2.05, 95% CI 1.80–2.33), or in Asian continent (2.46, 95% CI, 2.04–3.02) were at higher risk of TB than DM patients in high-income countries, in settings with TB incidence ≤50, or in Europe or USA and Canada, respectively (S1 Table).

In two prospective studies [8, 35], with microbiologically defined TB, and with blood testing for DM, patients were at 3.67–fold (95% CI 2.01–6.70) increased risk of TB. Overall, DM patients with microbiologically defined TB were at 3.03-fold (95% CI 2.31–3.98) increased risk of TB compared to 1.58–fold (95% CI 1.46–1.71) and 1.73–fold (95% CI 1.36–2.20) based on medical records or self-reported TB, respectively. Overall, blood tested DM patients were at 3.10–fold (95% CI 2.02–4.74) increased risk of TB compared to 1.60-fold (95% CI 1.18–2.17) and 1.95-fold (95% CI 0.90–4.25) based on medical records or self-reported DM, respectively (S1 Table).

Overall, DM patients with HbA1c ≥ 6.5%, FBG ≥ 120 mg/dl, or on insulin treatment, were at 1.87–fold (95% CI 1.19–2.93), 3.30-fold (95% CI 2.12–5.14), or 2.51–fold (95% CI 1.62–3.87) increased risk of TB, respectively (S2 Table).

In the four prospective (*p* = 0.495), 16 retrospective that reported RR, RRs, or HR (*p* = 0.439), three retrospective that reported an OR (*p* = 0.864), and three cross-sectional (*p* = 0.696) studies, reporting on the association between DM and TB, the Egger’s *t* statistic for asymmetry in the funnel plot indicated no evidence for the presence of a small-study effect (S2a, S2b, S2c and S2d Fig, respectively). However, in the 17 case-control studies, the Egger’s *t* statistic for asymmetry in the funnel plot indicated evidence for the presence of a small-study effect (*p* = 0.005) (S2e Fig).

With a summary RRs of 3.59 among DM patients in the four prospective studies, DM accounts for 72% of active TB cases among strictly DM patients (attributable risk fraction). In the six high-TB-burden countries (India, Indonesia, China, Nigeria, Pakistan, and South Africa), that accounted for 60% of new TB cases in 2015, 18%, 14%, 22%, 11%, 15%, and 15% of active TB cases in the entire population in these countries is attributed to DM, respectively. For the population attributable risk fraction, calculations are presented in S1 Text.

## Discussion

In this systematic review and meta-analysis of studies on the TB–DM association, we identified a strong positive association, but with substantial heterogeneity in effect size between studies. Stronger associations were noted among DM patients where TB was confirmed microbiologically, where DM ascertainment was based on blood testing (rather than self-report or medical records only), and among DM patients with uncontrolled blood glucose. This most comprehensive review and meta-analysis included 44 observational studies, compared to nine and 13 [13] studies in the previously published two reviews, one of which included a meta-analysis of prospective studies [13]. With this increase in published evidence, our meta-analysis confirmed the result of the earlier meta-analysis [13] and strengthened the evidence base for a strong association between DM and active TB. Our results demonstrated consistent evidence of a two- to four-fold increased risk of developing TB disease for DM patients compared to non-DM patients.

As a result of aging and increasing prevalence of major DM risk factors; particularly obesity and tobacco use [1, 67–69], it is projected that the number of individuals with DM will rise from 415 million in 2015 to 642 million by 2040 [3]. With the observed TB relative risk of 3.59 among DM patients in prospective studies, DM will therefore account for an increasing fraction of active TB cases in the entire population in the future. A frequent misperception is that chronic diseases such as DM are diseases of affluence [20, 70], in fact they are also common amongst poorer populations, where infectious diseases such as TB remain prevalent. Estimates suggest that the DM burden is increasing fastest in those regions where TB remains endemic [46]. From a public health perspective, it is of note that there are now more TB patients with concomitant DM than with HIV [71]. Given these findings, it may be challenging to control TB, particularly in settings that experience the double burden of the twin–epidemic of TB and DM. Robust public health intervention programs should consider tackling the underlying factors of DM such as lack of physical activity and obesity. As well as, programs to screen DM patients for TB alongside controlling blood glucose in TB patients to improve TB/DM treatment outcomes and to control this “twin epidemic”. Our findings strengthen the evidence base for how DM can impact upon the achievements of the WHO “*End TB Strategy*” [1].

The observed slight discrepancy in the summary estimate in the present and the previous meta-analysis [13] is partially due to the fact that one of the prospective studies [24] included in the previous meta-analysis was classified as retrospective in our review. Also, we used estimates from more confirmatory TB (bacterial culture confirmed rather than X-ray ascertained TB) and DM (HbA1c rather than FBG ascertained DM) ascertainment criteria that adjusted for the largest number of variables to pool strata-specific estimates, which in turn would produce more reliable association between TB and DM. In the previous meta-analysis [13], the estimate obtained from Kim et al., 1995 [8] was age-adjusted for all types of TB using a fixed-effect model, whereas we adjusted for sex for bacteriologically proven TB only, using a random-effects model. Moreover, we identified two more prospective studies [23, 35] that contributed 44% of the weight in our summary estimate. However, excluding one of these two studies, as DM and one-third of TB cases were ascertained by “self-report”, the summary estimate was 3.18 (95% CI 1.95–5.18), which is more comparable to that reported in the previous meta-analysis [13]. In the prospective studies, DM was mostly ascertained prior to the development of TB, suggesting that DM increases the risk of developing active TB, though some studies screened for DM at the time of TB diagnosis, and thus infection-related hyperglycemia could also explain some of the association.

We conducted sub-group sensitivity analyses to assess the heterogeneity in effect size. Several factors appeared to have contributed to this heterogeneity, including sampling methodology, study subjects, year of study, geographical location, exposure and outcome ascertainment methodology, variability within the specific subpopulation studied, sex and age-group representation in the sample, and publication bias. However, with the relatively small number of outcome measures according to study design, it was not possible to quantify the contribution of these sources of variation to the heterogeneity in the association through a meta-regression analysis.

All included studies were adjusted for at least age or sex, and estimates from majority of studies were also adjusted for different demographic and other potential confounders. This ensured that overall summary estimates were adjusted for at least the major confounding effects of age or sex. The strongest TB–DM association was observed from the four prospective studies [8, 23, 35, 36]. Data from almost three-quarters of included studies were representative of the general population. In studies reporting more than one adjusted estimate or strata-specific estimates, we included the estimates with more confirmatory ascertainment criteria for TB and/or DM, and that adjusted for the largest number of variables. This in turn produced summary estimates with lower potential of including false positive or false negative DM or TB cases. Overall, limiting the meta-analysis to studies judged as “potentially of low ROB” and excluding potentially duplicate studies did not change the direction nor magnitude of the association.

In the present review, the overall summary estimate in settings with TB incidence >50 cases per 100,000 person-year showed stronger association compared to that in settings with TB incidence ≤50 (S1 Table). This is in line with the findings of the previous meta-analysis [13]. Dobler et al., 2012 [47], hypothesized that the reason for the stronger association in settings with higher TB incidence could relate to the quality of diabetes management, assuming healthcare services may be poorer or harder to access in higher TB incidence settings.

We noticed a stronger association in blood-tested DM patients. DM patients with well-controlled glucose levels are less likely to be included when the definition of DM is based on blood glucose levels, which implies that hyperglycaemia rather than a DM diagnosis per se, increases the risk of TB [47]. DM patients suffer from immune system impairments, resulting in a lack of energy supply to immune cells, that subsequently increases virulence of infectious microorganisms [72–75]. These impairments weaken the immune system response to *Mycobacterium tuberculosis* [76–78]. This is supported by the observed stronger association in patients with uncontrolled blood glucose level (FBG ≥120 mg/dl or HbA1c ≥6.5%) (S2 Table).

There are several biological mechanisms that appear to alter the immune system and by which DM patients may develop TB [72–84]. High levels of insulin were associated with a decrease in T helper 1 (Th1) immunity through a reduction in the Th1 cell to T helper 2 (Th2) cell ratio and interferon-c (IFN-c) to interleukin-4 (IL-4) ratio [80]. Other studies showed that nonspecific IFN-c levels were significantly reduced in people with diabetes compared to people with no diabetes [81], and that levels of IFN-c were negatively correlated with levels of HbA1c [82]. Neutrophils in people with diabetes were found with a lack in chemotaxis and oxidative killing potential compared to non-diabetic controls [83]. Leukocyte bactericidal activity was found to be reduced in people with diabetes, especially those with poor glucose control [84]. These observed immunologic alterations seen in people with diabetes have also been supported in experimental animal studies. Diabetic mice experimentally infected with *Mycobacterium tuberculosis* have higher bacterial loads compared to euglycemic mice [85, 86] with significantly lower production of IFN-c and interleukin-12 and fewer T cells [86].

Several of the included studies had methodological weaknesses. Eight studies [5, 12, 23, 52, 54, 55, 61, 62] relied on “self-reported” DM and four studies [12, 23, 54, 62] relied on “self-reported” TB. Studies that utilized blood tests to define DM may also have reported stronger associations between DM and TB, since they can identify undiagnosed DM [13], which is common in many low–and middle–income countries. In studies that relied on “self-reported” DM, subjects with controlled blood glucose (euglycemic) would be “misclassified” as DM patients. This assumption is supported in our sensitivity analyses (S1 Table). There is an additional potential misclassification of TB and DM cases as studies often used routinely collected data without validation using laboratory tests [38]. For instance, a single HbA1c measurement might misclassify individuals as either DM or non-DM patients. It is recommended that DM diagnosis should be confirmed with a repeat HbA1c test, unless clinical symptoms and plasma glucose levels >11.1mmol/l (200 mg/dl) are present [87]. Missing adjustment for potential confounders is also a noteworthy limitation. In six studies [16, 45–48, 76], estimates were adjusted only for age and/or sex. Individual studies that controlled for the influence of age, sex, and smoking [35, 53, 58] produced stronger estimates than those controlled for age and sex [20, 43, 47]. Biased estimates on the TB–DM association may have occurred in studies among patients receiving dialysis [24, 41] or among subjects from specialty clinics or hospitals rather than the general population [88]. In almost all case-control studies, sampling of cases and/or controls was based on non-probability sampling. Studies using hospital-based controls reported weaker estimates for the association [13].

DM can affect different aspects of TB natural history and treatment outcomes, and therefore can impact TB transmission dynamics. An ongoing study has identified seven epidemiologically-relevant plausible effects for DM on TB natural history, and three for DM on TB treatment outcomes [89]. Our study, however, was focused on one major aspect of the TB–DM synergy, that of the association between DM status and active TB disease diagnosis—we did not assess other aspects of this synergy such as effects of DM on TB infection acquisition, TB reactivation among those latently infected, TB infectiousness, or TB treatment outcomes. A recent review, for example, reported that DM increases risk of latent TB by 1.18–fold [14], though with substantial heterogeneity across studies, and other studies have demonstrated major effects for DM on TB treatment outcomes [15–17]. Comprehensive and granular characterization and quantification of the diverse effects of DM on TB is essential for a proper understanding and estimation of the impact of DM on TB epidemiology.

In most of included studies, type of DM was unclear, thereby limiting our ability to assess the association by DM type (1, 2, or both). Having said so, in the three studies that assessed the association of TB with type 1 DM [9, 48, 51], the effect size was comparable to that seen for type 2 DM studies. HIV/AIDS is a strong risk factor for TB [90], but only a fraction of included studies controlled for its effect in their assessment of the association. This may not affect appreciably our results, as HIV prevalence is very low in nearly all countries where the association was assessed. Age is another confounding factor for the TB–DM association, and nearly all studies controlled for this factor. Our study was focused on the overall effect of DM on TB disease, and we did not provide a pooled effect size stratified by age. Despite these limitations, our review and meta-analyses compiled and summarized important data and critically provided narrative information from a large number of studies that reported on the TB–DM association.

## Conclusions

Our systematic review and meta-analysis demonstrated consistent evidence of a substantially increased risk of TB disease among people with DM. This evidence was based on data from studies using different designs and reported from six continents. DM patients with uncontrolled blood glucose (measured by higher FBG or HbA1c) appeared to be at higher risk of active TB than patients with controlled DM. Efforts to halt the burgeoning DM epidemic would have an accompanied benefit of alleviating the global burdens of DM and TB. The burgeoning epidemic of DM is likely to impact upon the achievements of the WHO “*End TB Strategy*”. Our findings inform strategy planning of health service provision and implementation of effective prevention programs to control the “twin epidemic” of DM and TB.

## Supporting information

S1 Table Summary findings of the meta-analyses for the sensitivity analyses of the association between DM and active TB in 44 studies, according to study design and overall. (DOCX)

S2 Table Estimates and summary estimates of the association between DM and active TB, according to DM ascertainment in blood-tested patients and study design. (DOCX)

S1 Fig PRISMA checklist. (DOCX)

S2 Fig Funnel plots assessing the risk of publication bias according to study design. (DOCX)

S1 Box Data sources and search criteria for systematically reviewing literature reporting on active tuberculosis (TB) and diabetes mellitus (DM) association. (DOCX)

S2 Box Criteria used to assess quality of included studies. (DOCX)

S1 Text Calculation of attributable risk fraction of TB among DM patients and population attributable risk fraction of TB due to DM. (DOCX)
