# Supplementary material for: Association between diabetes mellitus and active tuberculosis: A systematic review and meta-analysis
Source: PLoS One. 2017 Nov 21;12(11):e0187967. doi: 10.1371/journal.pone.0187967 (PMC5697825; doi:10.1371/journal.pone.0187967)
Supplement: S1 Table — (DOCX) [file pone.0187967.s001.docx]

**Table S1:** Summary findings of the meta-analyses for the sensitivity analyses of the association between DM and active TB in 44 studies, according to study design and overall

|  | **Studies** | **Range of estimates** | **Pooled effect estimate** | |  | **Heterogeneity measures** | | |
| --- | --- | --- | --- | --- | --- | --- | --- | --- |
|  | Total N | Lowest–Highest | Summary estimate | 95% CI |  | *Q (p-value)*^1^ | *τ^2^* ^2^ | *I*^2 3^ |
| **Potentially, low ROB** |  |  |  |  |  |  |  |  |
| Prospective | 4 | 1.86˗ 7.58 | 3.59 | 2.25˗5.73 |  | 13.55 (*p* = 0.004) | 0.1619 | 77.9% |
| Retrospective (RR, RRs, HR) | 15 | 1.00˗ 4.23 | 1.55 | 1.40˗1.72 |  | 65.48 (*p* < 0.0001) | 0.0224 | 78.6% |
| Retrospective (OR) | 2 | 1.42˗ 3.59 | 1.62 | 0.86˗3.07 |  | 1.25 (*p* = 0.263) | 0.0866 | 19.6% |
| Case-control | 13 | 1.16˗ 5.20 | 1.97 | 1.61˗2.42 |  | 58.86 (*p* < 0.0001) | 0.0838 | 79.6% |
| Cross-sectional^4^ | 1 | 3.17 | 3.17 | 1.14˗8.84 |  | –^8^ | –^8^ | –^8^ |
| Other^4^ | 1 | 6.00 | 6.00 | 5.00–7.20 |  | –^8^ | –^8^ | –^8^ |
| **Overall**^5^ | **36** | **1.00˗ 7.58** | **2.00** | **1.77˗2.27** |  | **431.38 (*p* < 0.0001)** | **0.0959** | **91.9%** |
| **Among general population**^6^ |  |  |  |  |  |  |  |  |
| Prospective (RRs, HR) | 3 | 2.69˗7.58 | 4.22 | 2.46˗7.25 |  | 9.41 (*p* < 0.0001) | 0.0249 | 81.6% |
| Retrospective (RR, RRs, HR) | 11 | 1.00˗4.23 | 1.60 | 1.40˗1.82 |  | 57.63 (*p* = 0.001) | 0.0252 | 82.6% |
| Retrospective (OR)^4^ | 1 | 1.81 | 1.81 | 1.37˗2.39 |  | –^8^ | –^8^ | –^8^ |
| Case-control | 14 | 1.18˗5.20 | 2.13 | 1.69˗2.68 |  | 66.15 (*p* < 0.0001) | 0.1126 | 80.3% |
| Cross-sectional | 3 | 1.40˗3.17 | 1.70 | 1.28˗2.24 |  | 2.81 (*p* = 0.245) | 0.0184 | 28.9% |
| Other^4^ | 1 | 6.00 | 6.00 | 5.00–7.20 |  | –^8^ | –^8^ | –^8^ |
| **Overall**^5^ | **33** | **1.00˗7.58** | **2.12** | **1.82˗2.48** |  | **367.97 (*p* < 0.0001)** | **0.1224** | **92.1%** |
| **Potentially duplicate data** |  |  |  |  |  |  |  |  |
| Retrospective (RR, RRs, HR) |  |  |  |  |  |  |  |  |
| No^7^ | 10 | 1.00˗3.07 | 1.60 | 1.41˗1.81 |  | 23.16 (*p* = 0.006) | 0.0157 | 61.6% |
| Yes | 6 | 1.25˗4.23 | 1.50 | 1.26˗1.80 |  | 21.49 (*p* = 0.001) | 0.0308 | 76.7% |
| Retrospective (OR) |  |  |  |  |  |  |  |  |
| No | 2 | 1.81˗3.59 | 1.85 | 1.40˗2.43 |  | 0.70 (*p* = 0.402) | 0.0000 | 0.0% |
| Yes^4^ | 1 | 1.42 | 1.42 | 0.96˗2.10 |  | –^8^ | –^8^ | –^8^ |
| **Overall**^5^ |  |  |  |  |  |  |  |  |
| **No^7^** | **12** | **1.00˗3.59** | **1.63** | **1.45˗1.82** |  | **25.48 (*p* = 0.006)** | **0.0157** | **56.3%** |
| **Yes** | **7** | **1.25˗4.23** | **1.48** | **1.26˗1.75** |  | **21.59 (*p* = 0.001)** | **0.0271** | **72.2%** |
| **Country income level**^9^ |  |  |  |  |  |  |  |  |
| Low- & middle-income |  |  |  |  |  |  |  |  |
| Prospective | 2 | 2.24˗2.69 | 2.54 | 1.94˗3.33 |  | 0.38 (*p* = 0.540) | 0.0000 | 0.0% |
| Retrospective (RR, RRs, HR)^4^ | 1 | 2.43 | 2.43 | 0.84˗7.00 |  | –^8^ | –^8^ | –^8^ |
| Retrospective (OR)^4^ | 1 | 3.59 | 3.59 | 0.74˗17.35 |  | –^8^ | –^8^ | –^8^ |
| Cross-sectional (OR)^4^ | 1 | 3.17 | 3.17 | 1.14˗8.83 |  | –^8^ | –^8^ | –^8^ |
| Other^4^ | 1 | 6.00 | 6.00 | 5.00˗7.20 |  | –^8^ | –^8^ | –^8^ |
| Case-control | 3 | 2.13˗ 4.70 | 2.95 | 1.97˗4.39 |  | 4.87 (*p* = 0.088) | 0.0733 | 58.9% |
| **Overall**^5^ | **9** | **2.13˗ 6.00** | **3.16** | **2.20˗4.53** |  | **43.20 (*p* < 0.0001)** | **0.2066** | **81.5%** |
| High-income countries |  |  |  |  |  |  |  |  |
| Prospective | 2 | 4.97˗7.58 | 5.17 | 3.88˗6.87 |  | 0.70 (*p* = 0.404) | 0.0000 | 0.0% |
| Retrospective (RR, RRs, HR) | 15 | 1.00˗4.23 | 1.54 | 1.39˗1.71 |  | 64.70 (*p* < 0.0001) | 0.0327 | 78.4% |
| Retrospective (OR) | 2 | 1.81˗1.42 | 1.67 | 1.33˗2.09 |  | 0.99 (*p* = 0.320) | 0.0000 | 0.0 |
| Cross-sectional (OR)^4^ | 1 | 1.40 | 1.40 | 0.99˗1.98 |  | –^8^ | –^8^ | –^8^ |
| Case-control | 14 | 1.16˗7.83 | 1.87 | 1.54˗2.28 |  | 47.98 (*p* < 0.0001) | 0.0685 | 72.9% |
| **Overall**^5^ | **34** | **1.00˗1.18** | **1.73** | **1.58–2.62** |  | **188.85 (*p* < 0.0001)** | **0.0390** | **82.5%** |
| **Background TB incidence**^10^ |  |  |  |  |  |  |  |  |
| Prospective |  |  |  |  |  |  |  |  |
| ≤50^4^ | 1 | 7.58 | 7.58 | 2.94˗19.49 |  | –^8^ | –^8^ | –^8^ |
| >50 | 3 | 2.24˗4.97 | 3.18 | 1.95˗5.18 |  | 10.98 (*p* = 0.004) | 0.1508 | 81.8% |
| Retrospective (RR, RRs, HR) |  |  |  |  |  |  |  |  |
| ≤50 | 7 | 1.00˗2.18 | 1.57 | 1.30˗1.90 |  | 20.06 (*p* = 0.003) | 0.03888 | 70.1% |
| >50 | 9 | 1.25˗4.23 | 1.51 | 1.33˗1.73 |  | 39.25 (*p* < 0.0001) | 0.0196 | 79.6% |
| Retrospective (OR) |  |  |  |  |  |  |  |  |
| ≤50^4^ | 1 | 1.81 | 1.81 | 1.37˗2.39 |  | –^8^ | –^8^ | –^8^ |
| >50 | 2 | 1.42˗3.59 | 1.63 | 0.86˗3.09 |  | 1.25 (*p* = 0.263) | 0.0866 | 20.1% |
| Cross-sectional (OR) |  |  |  |  |  |  |  |  |
| ≤50^4^ | 1 | 1.40 | 1.40 | 1.00˗2.00 |  | –^8^ | –^8^ | –^8^ |
| >50^4^ | 1 | 3.17 | 3.17 | 1.14˗8.84 |  | –^8^ | –^8^ | –^8^ |
| Case-control |  |  |  |  |  |  |  |  |
| ≤50 | 9 | 1.16˗3.80 | 1.74 | 1.39˗2.24 |  | 24.42 (*p* = 0.002) | 0.0826 | 67.2% |
| >50 | 8 | 1.46˗7.83 | 2.76 | 1.81˗4.20 |  | 53.44 (*p* < 0.0001) | 0.2626 | 86.9% |
| Other |  |  |  |  |  |  |  |  |
| ≤50^4^ | 1 | 6.00 | 6.00 | 5.00˗7.20 |  | –^8^ | –^8^ | –^8^ |
| **Overall**^5^ |  |  |  |  |  |  |  |  |
| **≤50** | **20** | **1.00˗7.58** | **1.89** | **1.36˗2.41** |  | **56.51 (*p* < 0.0001)** | **0.0507** | **68.1%** |
| **>50** | **23** | **1.25˗7.83** | **2.05** | **1.80˗2.33** |  | **233.75 (*p* < 0.0001)** | **0.2446** | **91.9%** |
| **Geographical region** |  |  |  |  |  |  |  |  |
| Prospective |  |  |  |  |  |  |  |  |
| Asia | 3 | 2.24˗4.97 | 3.18 | 1.95˗5.18 |  | 10.98 (*p* = 0.004) | 0.1508 | 81.8% |
| USA or Canada^4^ | 1 | 7.58 | 7.58 | 2.94˗19.49 |  | –^8^ | –^8^ | –^8^ |
| Retrospective (RR, RRs, HR) |  |  |  |  |  |  |  |  |
| Asia | 9 | 1.25˗4.23 | 1.51 | 1.33˗1.73 |  | 39.25 (*p* < 0.0001) | 0.0196 | 79.6% |
| Europe | 3 | 1.30˗2.02 | 1.55 | 1.29˗1.87 |  | 4.09 (*p* = 0.130) | 0.0138 | 51.1% |
| USA or Canada | 3 | 1.00˗1.76 | 1.55 | 0.82˗2.93 |  | 13.37 (*p* = 0.001) | 0.2347 | 85.0% |
| Australia^4^ | 1 | 1.49 | 1.49 | 1.05˗2.11 |  | –^8^ | –^8^ | –^8^ |
| Retrospective (OR) |  |  |  |  |  |  |  |  |
| Asia | 2 | 1.42˗3.59 | 1.63 | 0.86˗3.09 |  | 1.25 (*p* = 0.263) | 0.0866 | 20.1% |
| USA or Canada^4^ | 1 | 1.81 | 1.81 | 1.37˗2.39 |  | –^8^ | –^8^ | –^8^ |
| Cross-sectional (OR) |  |  |  |  |  | –^8^ | –^8^ | –^8^ |
| Asia^4^ | 1 | 3.17 | 3.17 | 1.40˗8.84 |  | –^8^ | –^8^ | –^8^ |
| USA or Canada^4^ | 1 | 1.40 | 1.40 | 0.99˗1.98 |  | –^8^ | –^8^ | –^8^ |
| Multicenter (43 countries)^4^ | 1 | 1.81 | 1.81 | 1.37˗2.39 |  | –^8^ | –^8^ | –^8^ |
| Case-control |  |  |  |  |  |  |  |  |
| Asia | 4 | 1.46˗4.70 | 2.43 | 1.29˗4.58 |  | 29.75 (*p* < 0.0001) | 0.3582 | 89.9% |
| Europe | 3 | 1.18˗3.80 | 2.15 | 0.91˗5.04 |  | 20.24 (*p* < 0.0001) | 0.4941 | 90.1% |
| USA or Canada | 7 | 1.16˗5.20 | 1.65 | 1.41˗1.94 |  | 5.73 (*p* = 0.455) | 0.0000 | 0.0% |
| Russia^4^ | 1 | 7.83 | 7.83 | 2.37˗25.89 |  | –^8^ | –^8^ | –^8^ |
| Africa^4^ | 1 | 2.13 | 2.13 | 1.37˗3.31 |  | –^8^ | –^8^ | –^8^ |
| Latin America^4^ | 1 | 28.0 | 2.80 | 1.96˗4.01 |  | –^8^ | –^8^ | –^8^ |
| Other |  |  |  |  |  |  |  |  |
| Latin America^4^ | 1 | 6.00 | 6.00 | 5.00˗7.20 |  | –^8^ | –^8^ | –^8^ |
| **Overall**^5^ |  |  |  |  |  |  |  |  |
| **Asia**^44^ | **12** | **1.46˗4.97** | **2.46** | **2.04˗3.02** |  | **107.84 (*p* < 0.0001)** | **0.0648** | **89.8%** |
| **Europe** | **6** | **1.18˗3.80** | **1.71** | **1.33˗2.20** |  | **24.85 (*p* < 0.0001)** | **0.0659** | **79.9%** |
| **USA or Canada** | **13** | **1.00˗7.58** | **1.73** | **1.43˗2.10** |  | **30.68 (*p* = 0.002)** | **0.0613** | **60.9%** |
| **TB ascertainment** |  |  |  |  |  |  |  |  |
| Microbiologically |  |  |  |  |  |  |  |  |
| Prospective | 2 | 2.69˗4.97 | 3.67 | 2.01˗6.70 |  | 7.40 (*p* = 0.007) | 0.1629 | 86.5% |
| Retrospective (RR, RRs, HR) | 3 | 1.76˗3.07 | 2.44 | 1.31˗4.52 |  | 0.50 (*p* = 0.779) | 0.0000 | 0.0% |
| Retrospective (OR)^4^ | 1 | 3.59 | 3.59 | 0.74˗17.35 |  | –^8^ | –^8^ | –^8^ |
| Case-control | 9 | 1.16˗7.83 | 2.86 | 2.25˗3.64 |  | 20.18 (*p* = 0.010) | 0.1499 | 60.4% |
| Cross-sectional^4^ | 1 | 3.17 | 3.17 | 1.14˗8.83 |  | –^8^ | –^8^ | –^8^ |
| Other^4^ | 1 | 6.00 | 6.00 | 5.00˗7.20 |  | –^8^ | –^8^ | –^8^ |
| **Overall**^5^ | **17** | **1.16˗7.83** | **3.03** | **2.31˗3.98** |  | **63.09 (*p* < 0.0001)** | **0.1944** | **75.0%** |
| Medical records |  |  |  |  |  |  |  |  |
| Prospective | 2 | 2.24˗7.58 | 3.84 | 1.17˗12.58 |  | 5.05 (*p* = 0.025) | 0.5958 | 80.2% |
| Retrospective (RR, RRs, HR) | 13 | 1.00˗4.23 | 1.53 | 1.38˗1.70 |  | 62.48 (*p* < 0.0001) | 0.0220 | 80.8% |
| Retrospective (OR) | 2 | 1.42˗1.81 | 1.67 | 1.33˗2.09 |  | 0.99 (*p* = 0.320) | 0.0000 | 0.0% |
| Case-control | 7 | 1.18˗5.20 | 1.62 | 1.34˗1.95 |  | 21.21 (*p* = 0.002) | 0.0352 | 71.7% |
| **Overall**^5^ | **24** | **1.00˗7.58** | **1.58** | **1.46˗1.71** |  | **100.26 (*p* < 0.0001)** | **0.0200** | **77.1%** |
| Self-reported |  |  |  |  |  |  |  |  |
| Cross-sectional | 2 | 1.40˗1.81 | 1.63 | 1.27˗2.08 |  | 1.28 (*p* = 0.257) | 0.0073 | 22.1% |
| Case-control^4^ | 1 | 2.31 | 2.31 | 1.36˗3.93 |  | –^8^ | –^8^ | –^8^ |
| **Overall**^5^ | **3** | **1.40˗2.31** | **1.73** | **1.36˗2.20** |  | **2.67 (*p* = 0.263)** | **0.0118** | **25.1%** |
| **DM status ascertainment** |  |  |  |  |  |  |  |  |
| Blood testing |  |  |  |  |  |  |  |  |
| Prospective | 3 | 2.24˗4.97 | 3.18 | 1.95˗5.18 |  | 10.98 (*p* = 0.004) | 0.1508 | 81.8% |
| Retrospective (RR, RRs, HR)^4^ | 1 | 1.30 | 1.30 | 1.03˗1.64 |  | –^8^ | –^8^ | –^8^ |
| Case-control | 4 | 2.13˗5.20 | 3.03 | 2.10˗4.38 |  | 5.51 (*p* = 0.138) | 0.0603 | 45.6% |
| Cross-sectional^4^ | 1 | 3.17 | 3.17 | 1.14˗8.83 |  | –^8^ | –^8^ | –^8^ |
| Other^4^ | 1 | 6.00 | 6.00 | 5.00˗7.20 |  | –^8^ | –^8^ | –^8^ |
| **Overall**^5^ | **10** | **1.30˗6.00** | **3.10** | **2.02˗4.74** |  | **119.98 (*p* < 0.0001)** | **0.3928** | **92.5%** |
| Medical records |  |  |  |  |  |  |  |  |
| Retrospective (RR, RRs, HR) | 14 | 1.00˗4.23 | 1.56 | 1.10˗2.23 |  | 63.65 (*p* < 0.0001) | 0.0229 | 79.6% |
| Retrospective (OR) | 3 | 1.42˗3.59 | 1.69 | 1.35˗2.12 |  | 1.88 (*p* = 0.391) | 0.0000 | 0.0% |
| Case-control | 8 | 1.18˗3.43 | 1.75 | 1.42˗2.16 |  | 33.22 (*p* < 0.0001) | 0.0584 | 78.9% |
| **Overall**^5^ | **25** | **1.00˗4.23** | **1.60** | **1.18˗2.17** |  | **100.23 (*p* < 0.0001)** | **0.0202** | **76.1%** |
| Self-reported |  |  |  |  |  |  |  |  |
| Prospective^4^ | 1 | 7.58 | 7.58 | 2.94˗19.49 |  | –^8^ | –^8^ | –^8^ |
| Retrospective (RR, RRs, HR)^4^ | 1 | 2.43 | 2.43 | 0.84˗7.01 |  | –^8^ | –^8^ | –^8^ |
| Case-control | 4 | 1.16˗2.38 | 1.87 | 1.32˗2.64 |  | 2.81 (*p* < 0.422) | 0.0000 | 0.0% |
| Cross-sectional^\|^ | 2 | 1.40˗1.81 | 1.63 | 1.27˗2.08 |  | 1.28 (*p* = 0.257) | 0.0073 | 22.1% |
| **Overall**^5^ | **8** | **1.16˗7.58** | **1.95** | **0.90˗4.25** |  | **14.06 (*p* = 0.050)** | **0.0785** | **50.2%** |
| ^1^ Q: Cochran Q statistic is a measure assessing the existence of heterogeneity in estimates of association between TB and DM.  ^2^ *τ^2^*: the estimated between-study variance in the true TB and DM association estimates. The *τ^2^* is for the variance of beta not for the back-transformed estimate.  ^3^ *I*^2^: a measure assessing the magnitude of between-study variation that is due to differences in TB and DM association estimates across studies rather than chance.  ^4^ Only one study fell in this category. Summary estimate represents the study reported estimate.  ^5^ Overall estimate including risk ratios, rate ratios, hazard ratios, and odds ratios, that is regardless of the measure of association and study design. Background incidence rate of TB did not exceed 2 per 100 person-year in studies estimating an OR, therefore it is reasonable to assume that TB is sufficiently rare so that the ORs would estimate the risk ratios.[1] Pooled estimate was implemented using a random-effects model.  ^6^ Excluding studies among specific clinical populations such as renal dialysis and kidney transplant recipients.  ^7^ Excluding 6 potentially duplicate studies in Taiwan while including only the most recent potentially duplicate study with the largest number of study subjects.[2]  ^8^ Meta-analysis was not conduced given the insufficient number of studies (one study).  ^9^ Based on the classification provided by the World Bank.[3]  ^10^ TB incidence in the background population per 100,000 person-year.  RRs: relative risk; RR: rate ratio; OR: odds ratio; HR: hazard ratio; CI: confidence interval; ROB: risk of bias | | | | | | | | |

**References**

1. Greenland S, Thomas DC. On the Need for the Rare Disease Assumption in Case-Control Studies. American Journal of Epidemiology. 1982;116(3):547-53. PubMed PMID: WOS:A1982PH07800015.

2. Chung WS, Lin CL, Hung CT, Chu YH, Sung FC, Kao CH, et al. Tuberculosis increases the subsequent risk of acute coronary syndrome: a nationwide population-based cohort study. International Journal of Tuberculosis and Lung Disease. 2014;18(1):79-83. doi: 10.5588/ijtld.13.0288. PubMed PMID: WOS:000329483000016.

3. Bank W. Country Income Groups. Available: <http://chartsbin.com/view/2438>. Accessed on October 11 2016. 2016.
