# Supplementary material for: Association between diabetes mellitus and active tuberculosis: A systematic review and meta-analysis
Source: PLoS One. 2017 Nov 21;12(11):e0187967. doi: 10.1371/journal.pone.0187967 (PMC5697825; doi:10.1371/journal.pone.0187967)
Supplement: S2 Table — (DOCX) [file pone.0187967.s002.docx]

**S2 Table:** Estimates and summary estimates of the association between DM and active TB, according to DM ascertainment in blood-tested patients and study design.

|  | **Effect estimate of those blood-tested as reported in studies** | | | |  |
| --- | --- | --- | --- | --- | --- |
|  | HbA1c indicative of controlled DM.  Estimate (95% CI) | HbA1c indicative of uncontrolled DM.  Estimate (95% CI) | FBG indicative of uncontrolled DM.  Estimate (95% CI) | Insulin users.  Estimate (95% CI) | Measure indicative of uncontrolled DM.^1^  Estimate (95% CI) |
| **Prospective studies** |  |  |  |  |  |
| Leung et al (2008)[1] | < 7%: 0.86 (0.42–1.75) | ≥ 7%: 2.69 (1.94–3.72) | – | – | 2.69 (1.94–3.72) |
| John et al (2001)[2] | – | – | ≥120 mg/dl: 2.24 (1.38–3.65) | – | 2.24 (1.38–3.65) |
| **Overall** |  |  |  |  | **2.54 (1.94–3.33)** |
| **Retrospective studies** |  |  |  |  |  |
| Pealing et al (2015)[3] | ≤ 6.5%: 1.34 (0.88–2.05) | 6.5–7.5%: 1.14 (0.76–1.70)  > 7.5%: 1.50 (1.04–2.17) | – | – | 1.32 (1.01–1.74)^‡^ |
| Dobler et al (2012)[4] | – | – | – | 2.44 (1.37–4.34) | 2.44 (1.37–4.34) |
| Baker et al (2012)[5] | – | – | – | 2.60 (1.34–5.03) | 2.60 (1.34–5.03) |
| **Overall** |  |  |  |  | **2.12 (1.49–3.00)** |
| **Case-control studies** |  |  |  |  |  |
| Alisjahbana et al (2006)[6] | – | – | ≥ 126 mg/dl: 4.70 (2.70–8.10) | – | 4.70 (2.70–8.10) |
| Mori et al (1992)[7] | – | – | ≥ 7.8 mmol/l (≥140 mg/dl):  5.20 (1.22–22.10) | – | 5.20 (1.22–22.10) |
| Viney et al (2015)[8] | – | ≥ 6.5%: 2.80 (2.00–4.10) |  | – | 2.80 (2.00–4.10) |
| Leegaard et al (2011)[9] | < 7%: 0.91 (0.51–1.63) | = 7–7.9%: 1.05 (0.41–2.66)  ≥ 8%: 1.19 (0.61–2.30) |  | – | 1.14 (0.66–1.96)^‡^ |
| **Overall** |  |  |  |  | **2.72 (1.42–5.21)** |
| **Cross**–**sectional studies** |  |  |  |  |  |
| Wang et al (2013)[10] | – | – | ≥ 7 mmol/l (≥ 126 mg/dl):  3.17 (1.14–8.84) | – | 3.17 (1.14–8.84) |
| **Overall**^2^ | **< 7%**  **1.11 (0.81–1.51)** | **≥ 6.5%**  **1.87 (1.19–2.93)** | **≥ 120 mg/dl**  **3.30 (2.12–5.14)** | **Insulin users**  **2.51 (1.62–3.87)** | **2.37 (1.75–3.19)** |

^1^ DM patients identified as with an uncontrolled DM status regardless of the measurement method.

^†^ Overall estimate including risk ratios, rate ratios, hazard ratios, and odds ratios, that is regardless of the measure of association and study design. Background incidence rate of TB did not exceed 2 per 100 person-year in studies estimating an OR, therefore it is reasonable to assume that TB is sufficiently rare so that the ORs would estimate the risk ratios.[11] Pooled estimate was implemented using a random-effects model.

^‡^ Study-specific estimates were pooled using a random-effects model.

FBG: fasting blood glucose; HbA1c: Glycated hemoglobin is a form of hemoglobin that is measured primarily to identify the three-month average plasma glucose concentration.

**References**

1. Leung CC, Lam TH, Chan WM, Yew WW, Ho KS, Leung GM, et al. Diabetic control and risk of tuberculosis: A cohort study. American Journal of Epidemiology. 2008;167(12):1486-94. doi: 10.1093/aje/kwn075. PubMed PMID: WOS:000256755900012.

2. John GT, Shankar V, Abraham AM, Mukundan U, Thomas PP, Jacob CK. Risk factors for post-transplant tuberculosis. Kidney International. 2001;60(3):1148-53. doi: DOI 10.1046/j.1523-1755.2001.0600031148.x. PubMed PMID: WOS:000170668100034.

3. Pealing L, Wing K, Mathur R, Prieto-Merino D, Smeeth L, Moore DAJ. Risk of tuberculosis in patients with diabetes: population based cohort study using the UK Clinical Practice Research Datalink. Bmc Medicine. 2015;13. doi: ARTN 135

10.1186/s12916-015-0381-9. PubMed PMID: WOS:000356483400001.

4. Dobler CC, Flack JR, Marks GB. Risk of tuberculosis among people with diabetes mellitus: an Australian nationwide cohort study. Bmj Open. 2012;2(1). doi: ARTN e000666

10.1136/bmjopen-2011-000666. PubMed PMID: WOS:000315037200075.

5. Baker MA, Lin HH, Chang HY, Murray MB. The Risk of Tuberculosis Disease Among Persons With Diabetes Mellitus: A Prospective Cohort Study. Clinical Infectious Diseases. 2012;54(6):818-25. doi: 10.1093/cid/cir939. PubMed PMID: WOS:000300790900015.

6. Alisjahbana B, van Crevel R, Sahiratmadja E, den Heijer M, Maya A, Istriana E, et al. Diabetes mellitus is strongly associated with tuberculosis in Indonesia. International Journal of Tuberculosis and Lung Disease. 2006;10(6):696-700. PubMed PMID: WOS:000237771100017.

7. Mori MA, Leonardson G, Welty TK. The Benefits of Isoniazid Chemoprophylaxis and Risk-Factors for Tuberculosis among Oglala Sioux Indians. Archives of Internal Medicine. 1992;152(3):547-50. doi: DOI 10.1001/archinte.152.3.547. PubMed PMID: WOS:A1992HH85800013.

8. Viney K, Cavanaugh J, Kienene T, Harley D, Kelly PM, Sleigh A, et al. Tuberculosis and diabetes mellitus in the Republic of Kiribati: a case-control study. Trop Med Int Health. 2015;20(5):650-7. doi: 10.1111/tmi.12462. PubMed PMID: WOS:000352534600010.

9. Leegaard A, Riis A, Kornum JB, Prahl JB, Thomsen VO, Sorensen HT, et al. Diabetes, Glycemic Control, and Risk of Tuberculosis A population-based case-control study. Diabetes Care. 2011;34(12):2530-5. doi: 10.2337/dc11-0902. PubMed PMID: WOS:000298122900009.

10. Wang QZ, Ma AG, Han XX, Zhao SL, Cai J, Ma YB, et al. Prevalence of Type 2 Diabetes among Newly Detected Pulmonary Tuberculosis Patients in China: A Community Based Cohort Study. Plos One. 2013;8(12). doi: ARTN e82660

10.1371/journal.pone.0082660. PubMed PMID: WOS:000328740300051.

11. Greenland S, Thomas DC. On the Need for the Rare Disease Assumption in Case-Control Studies. American Journal of Epidemiology. 1982;116(3):547-53. PubMed PMID: WOS:A1982PH07800015.
