# Supplementary material for: Association between diabetes mellitus and active tuberculosis: A systematic review and meta-analysis
Source: PLoS One. 2017 Nov 21;12(11):e0187967. doi: 10.1371/journal.pone.0187967 (PMC5697825; doi:10.1371/journal.pone.0187967)
Supplement: S1 Fig — (DOCX) [file pone.0187967.s003.docx]

**S1 Figure:** Preferred Reporting Items for Systematic Reviews and Meta-analyses (PRISMA) 2009 checklist [1].

| **Section/topic** | **#** | **Checklist item** | **Reported in main text on** |
| --- | --- | --- | --- |
| **TITLE** | | |  |
| Title | 1 | Identify the report as a systematic review, meta-analysis, or both.  The title page identifies this report as a systematic review and meta-analysis | p. 1 |
| **ABSTRACT** | | |  |
| Structured summary | 2 | Provide a structured summary including, as applicable: background; objectives; data sources; study eligibility criteria, participants, and interventions; study appraisal and synthesis methods; results; limitations; conclusions and implications of key findings; systematic review registration number.  Providing a structured abstract is not applicable as per Plos One’s Journal guidelines. However, an unstructured summary covers background, implemented methods, findings, conclusions and implication of key findings. | p. 2 |
| **INTRODUCTION** | | |  |
| Rationale | 3 | Describe the rationale for the review in the context of what is already known.  In the first and second paragraphs in the “Introduction” section we rationalized the reason behind conducting this systematic review and meta-analysis. | p. 3 |
| Objectives | 4 | Provide an explicit statement of questions being addressed with reference to participants, interventions, comparisons, outcomes, and study design (PICOS).  In the third paragraph in the “Introduction” section, we clarified the objective of conducting this systematic review and meta-analysis. | p. 4 |
| **METHODS** | | |  |
| Protocol and registration | 5 | Indicate if a review protocol exists, if and where it can be accessed (e.g., Web address), and, if available, provide registration information including registration number.  Not applicable. The review protocol was provided during the review process only. | NA |
| Eligibility criteria | 6 | Specify study characteristics (e.g., PICOS, length of follow-up) and report characteristics (e.g., years considered, language, publication status) used as criteria for eligibility, giving rationale.  We specified the exact inclusion and exclusion criteria of studies to be included in this systematic review in the “Methods” section, under the subtitle “Search strategy and selection criteria”. | p. 4 & 5 |
| Information sources | 7 | Describe all information sources (e.g., databases with dates of coverage, contact with study authors to identify additional studies) in the search and date last searched.  We described that the information sources included the Medline (from 1945 to December 22, 2015) and EMBASE (from 1980 to December 22, 2015) databases in the “Methods” section, under the subtitle “Search strategy and selection criteria”. | p. 4 |
| Search | 8 | Present full electronic search strategy for at least one database, including any limits used, such that it could be repeated.  We summarised the full electronic search strategy for the Medline and EMBASE databases in “Box S1 in the appendix” and detailed it in the “Methods” section, under the subtitle “Data extraction and quality assessment”. | p. 4 & 5  S1 Box |
| Study selection | 9 | State the process for selecting studies (i.e., screening, eligibility, included in systematic review, and, if applicable, included in the meta-analysis).  We summarised the process for selecting studies in “Figure 1” and detailed it in “Methods” section, under the subtitle “Data extraction and quality assessment”. | p. 4 & 5  Figure 1 |
| Data collection process | 10 | Describe method of data extraction from reports (e.g., piloted forms, independently, in duplicate) and any processes for obtaining and confirming data from investigators.  We described the method of data extraction in the “Methods” section, under the subtitle “Data extraction and quality assessment”. | p. 5 |
| Data items | 11 | List and define all variables for which data were sought (e.g., PICOS, funding sources) and any assumptions and simplifications made.  In priori, we defined all variables to be extracted. We detailed that in the “Methods” section, under the subtitle “Data extraction and quality assessment”. | p. 5 & 6 |
| Risk of bias in individual studies | 12 | Describe methods used for assessing risk of bias of individual studies (including specification of whether this was done at the study or outcome level), and how this information is to be used in any data synthesis.  We described the methods used for assessing risk of bias (ROB) of individual studies based on the Cochrane’s guidelines for conducting systematic reviews and other validated quality assessment tools. The domains used to assess the ROB are listed in “Box S2” in the appendix and detailed in the “Methods” section, under the subtitle “Data extraction and quality assessment”. | p. 6  S2 Box |
| Summary measures | 13 | State the principal summary measures (e.g., risk ratio, difference in means).  We stated that the principle summary measure was the strength of association between TB and DM measured in the form of pooled adjusted odds ratios, hazard ratios, relative risks, or rate ratios. This was done in the “Methods” section, under the subtitle “Statistical analysis”. | p. 6 & 7 |
| Synthesis of results | 14 | Describe the methods of handling data and combining results of studies, if done, including measures of consistency (e.g., I^2^) for each meta-analysis.  In the “Methods” section, under the subtitle “Statistical analysis”, we described that the adjusted estimates were pooled stratified by the study design and regardless of study design using random-effects models. We also estimated the I-squared (*I^2^*) as a measure of heterogeneity and Tau-squared (*τ*^2^) as a measure of the between-study variance of the true association between TB and DM. | p. 7 & 8 |
| Risk of bias across studies | 15 | Specify any assessment of risk of bias that may affect the cumulative evidence (e.g., publication bias, selective reporting within studies).  We assessed the presence of publication bias by examining the funnel plots using the Egger’s *t* statistic to examine asymmetry in the funnel plot. We presented the funnel plots in Figure S2 in the appendix and provided narrative information in the “Methods” section, under the subtitle “Statistical analysis”. | p. 7 |
| Additional analyses | 16 | Describe methods of additional analyses (e.g., sensitivity or subgroup analyses, meta-regression), if done, indicating which were pre-specified.  We performed additional analyses in the form of sensitivity analyses pooling adjusted estimates according to different study’s characteristics and TB/DM ascertainment methodology as detailed in the “Methods” section, under the subtitle “Statistical analysis”. | p. 7 & 8 |
| **RESULTS** | | |  |
| Study selection | 17 | Give numbers of studies screened, assessed for eligibility, and included in the review, with reasons for exclusions at each stage, ideally with a flow diagram.  We provided a flow diagram showing the number of studies screened, assessed for eligibility, and included in the review, with reasons for exclusions at each stage. Flow diagram is presented in “Figure 1”. | Figure 1 |
| Study characteristics | 18 | For each study, present characteristics for which data were extracted (e.g., study size, PICOS, follow-up period) and provide the citations.  We described the characteristics of the included studies in “Tables 1 and 2” and summarised those characteristics under the “Results” section. | p. 8 & 9  Tables 1 and 2 |
| Risk of bias within studies | 19 | Present data on risk of bias of each study and, if available, any outcome level assessment (see item 12).    We presented the data on risk of bias in “S1 & S2 Tables” and in “S2 Figure” with narrative information under the “Results” section. | S1 & S2 Tables, and S2 Figure |
| Results of individual studies | 20 | For all outcomes considered (benefits or harms), present, for each study: (a) simple summary data for each intervention group (b) effect estimates and confidence intervals, ideally with a forest plot.  We presented simple summary estimate according to study design in Table 3 and depicted those summary estimates in a forest plot in Figure 2. | p. 26 & 27 |
| Synthesis of results | 21 | Present results of each meta-analysis done, including confidence intervals and measures of consistency.  We presented summary estimate according to study design in Table 3 and depicted those summary estimates in a forest plot in Figure 2. | Table 3  Figure 2 |
| Risk of bias across studies | 22 | Present results of any assessment of risk of bias across studies (see Item 15).  We presented the results of assessment of risk of publication bias in “S2 Figure” and provided narrative information under the “Results” section in the main text. | p. 15  S2 Figure |
| Additional analysis | 23 | Give results of additional analyses, if done (e.g., sensitivity or subgroup analyses, meta-regression [see Item 16]).  We provided the results of sensitivity analyses including measures of heterogeneity in “Tables S1 and S2” in the appendix with summarised narrative information in the “Results” section in the main text. | p. 28    S1 & S2 Tables |
| **DISCUSSION** | | |  |
| Summary of evidence | 24 | Summarize the main findings including the strength of evidence for each main outcome; consider their relevance to key groups (e.g., healthcare providers, users, and policy makers).  The first paragraph in the “Discussion” section summarises the main findings of this systematic review. | p. 29 & 30 |
| Limitations | 25 | Discuss limitations at study and outcome level (e.g., risk of bias), and at review-level (e.g., incomplete retrieval of identified research, reporting bias).  We discussed the individual study’s limitations as well as the overall limitations of this systematic review in the second paragraph in the “Discussion” section. | p. 34 |
| Conclusions | 26 | Provide a general interpretation of the results in the context of other evidence, and implications for future research.  We provided general interpretation of the results in the context of other evidence, and implications in the last paragraph in the “Methods” section. | p. 26 |
| **FUNDING** | | |  |
| Funding | 27 | Describe sources of funding for the systematic review and other support (e.g., supply of data); role of funders for the systematic review.  This publication was made possible by NPRP grant number 7-627-3-167 from the Qatar National Research Fund (a member of Qatar Foundation). The statements made herein are solely the responsibility of the authors and the funder had no role in study design, data collection and analysis, decision to publish, or preparation of the manuscript. | P. 37 |

^*^Abbreviations: NA: Not applicable, p: page, SI: Supporting Information.

**References**

1. Moher D, Liberati A, Tetzlaff J, Altman DG, Group P. Preferred reporting items for systematic reviews and meta-analyses: the PRISMA statement. BMJ. 2009;339:b2535. doi: 10.1136/bmj.b2535. PubMed PMID: 19622551; PubMed Central PMCID: PMCPMC2714657.
