# Supplementary material for: Association between diabetes mellitus and active tuberculosis: A systematic review and meta-analysis
Source: PLoS One. 2017 Nov 21;12(11):e0187967. doi: 10.1371/journal.pone.0187967 (PMC5697825; doi:10.1371/journal.pone.0187967)
Supplement: S2 Fig — (DOCX) [file pone.0187967.s004.docx]

**(a)** Egger’s test: bias coefficient = 0.32; *t* = 0.80, *p* = 0.439) **(b)** Egger’s test: bias coefficient = 0.47; *t* = 0.83, *p* = 0.495)

**(c)** Egger’s test: bias coefficient = 0.15; *t* = 0.22, *p* = 0.864) **(d)** Egger’s test: bias coefficient = 0.42; *t* = 0.52, *p* = 0.696)

**(e)** Egger’s test: bias coefficient = 1.75; *t* = 3.29, *p* = 0.005)

**S2 Figures:** Funnel plots assessing the risk of publication bias of the (a) four prospective studies, (b) 16 retrospective studies that reported RR, RRs, or HR (c) three retrospective studies that reported an OR, (d) three cross–sectional studies, and (e) 17 case–control studies. Slope line in the funnel plots represents the fitted-regression line from the standard regression (Egger) test for small study-effects. Straight line in the funnel plots represents the pooled effect estimate using the fixed–effect model (not the null hypothesis of 0). RRs: relative risk; RR: rate ratio; OR: odds ratio; HR: hazard ratio.
