# Supplementary material for: Association between diabetes mellitus and active tuberculosis: A systematic review and meta-analysis
Source: PLoS One. 2017 Nov 21;12(11):e0187967. doi: 10.1371/journal.pone.0187967 (PMC5697825; doi:10.1371/journal.pone.0187967)
Supplement: S1 Box — (DOCX) [file pone.0187967.s005.docx]

***Search protocol***

**S1 Box.** Data sources and search criteria for systematically reviewing literature reporting on active tuberculosis (TB) and diabetes mellitus (DM) association.

The search strategy that had been used to identify studies of the TB-DM association:

1. TB AND DM.
2. TB AND risk factor(s) AND study design.
3. TB AND chronic disease(s).

**Medline**

**The final search protocol for Medline from 1945 to Dec 22, 2015 limited to studies on human subjects**

***Literature search string using variant Mesh and Text terms* *combined***

(("tuberculosis"[Mesh] OR "tuberculosis"[Text] OR "TB"[Text]) AND ("diabetes mellitus"[Mesh] OR "diabetes"[text] OR "DM"[Text] OR "glucose tolerance"[Text] OR "glucose intolerance"[Text] OR "glucose intolerance"[Mesh] OR "insulin resistance"[Text] OR "insulin resistance"[Mesh] OR "hyperglycemia"[Text] OR "hyperglycemia"[Mesh] OR "hypoglycemia"[text] OR "hypoglycemia"[Mesh]))

OR (("tuberculosis"[Mesh] OR "tuberculosis"[Text] OR "TB"[Text]) AND ("risk factors"[Text] OR "risk factors"[Mesh] OR "risk factor"[Text]) AND ("observational study"[Text] OR "observational studies"[Text] OR "cohort studies"[MeSh] OR "cohort studies"[Text] OR "case-control studies"[MeSh] OR "case-control studies"[Text] OR "cross-sectional studies"[MeSh] OR "cross-sectional studies"[Text] OR "epidemiologic studies"[MeSh] OR "epidemiologic studies"[Text] OR "follow-up studies"[MeSh] OR "follow-up studies"[Text] OR "longitudinal studies"[MeSh] OR "longitudinal studies"[Text] OR "prospective studies"[MeSh] OR "prospective studies"[Text] OR "retrospective studies"[MeSh] OR "retrospective studies"[Text] OR "cohort study"[Text] OR "case-control study"[Text] OR "cross-sectional study"[Text] OR "epidemiologic study"[Text] OR "follow-up study"[Text] OR "longitudinal study"[Text] OR "prospective study"[Text] OR "retrospective study"[Text]))

OR (("tuberculosis"[Mesh] OR "tuberculosis"[Text] OR "TB"[Text]) AND ("chronic diseases"[Text] OR "chronic disease"[Mesh] OR "chronic disease"[Text] OR "non-communicable diseases"[MeSh] OR "non-communicable disease"[Text]))

**EMBASE**

**The final search protocol for EMBASE from 1980 to Dec 22, 2015 limited to studies on human subjects**

***Literature search string using variant Emtree and Text terms combined***

((tuberculosis.mp. or exp tuberculosis/) AND (diabetes mellitus.mp. or exp diabetes mellitus/ OR diabetes.mp. or exp diabetes / or glucose tolerance.mp. or exp glucose tolerance/ or glucose intolerance.mp. or exp glucose intolerance/ or insulin resistance.mp. or insulin resistance/ or hypoglycemia.mp. or exp hypoglycemia/ or hyperglycemia.mp. or exp hyperglycemia/))

OR ((tuberculosis.mp. or exp tuberculosis/) AND (risk factor.mp. or exp risk factor/) AND (observational studies.mp. or exp observational study/ or Cohort studies.mp. or exp cohort analysis/ or case control studies.mp. or exp case control study/ or cross sectional studies.mp. or exp cross-sectional study/ or epidemiologic studies.mp. or exp epidemiology/ or follow-up studies.mp. or exp follow up/ or longitudinal studies.mp. or exp longitudinal study/ or prospective studies.mp. or exp prospective study/ or retrospective studies.mp. or exp retrospective study/))

OR ((tuberculosis.mp. or exp tuberculosis/) AND (Chronic diseases.mp. or exp chronic disease/ or non-communicable diseases.mp. or exp non communicable disease/))
