# Supplementary material for: Association between diabetes mellitus and active tuberculosis: A systematic review and meta-analysis
Source: PLoS One. 2017 Nov 21;12(11):e0187967. doi: 10.1371/journal.pone.0187967 (PMC5697825; doi:10.1371/journal.pone.0187967)
Supplement: S2 Box — (DOCX) [file pone.0187967.s006.docx]

**Quality assessment**

**S2 Box.** Criteria used to assess quality of included studies.

1. Response rate among selected/recruited subjects was ≥80%:^*^
2. Yes (0) No (0) Unclear
3. Study provided an adequate case definition for TB:^*^
4. Yes (0) No (0) Unclear
5. Study provided an adequate case definition for DM:^*^

(1) Yes (0) No (0) Unclear

1. Method used to ascertain DM was:^*^

(1) Blood testing, medical records, ICD-codes, physician diagnosis, or prescription of anti-DM medications

(0) Self-report/TB symptoms

(0) Unclear

1. Method used to ascertain TB:^*^

(1) Microbiology, medical records, ICD-codes, physician diagnosis, or prescription of anti-TB medications

(0) Self-report/TB symptoms

(0) Unclear

1. Same method of TB ascertainment was used for all study subjects: ^*^

(1) Yes (0) No (0) Unclear

1. Same method of DM ascertainment was used for all study subjects: ^*^

(1) Yes (0) No (0) Unclear

1. DM diagnosis pre-dated TB diagnosis: ^*^

(1) Yes (0) No (0) Unclear

1. Rigor of sampling methodology:^†^

(1) Probability-based

(0) Non-probability-based

(0) Unclear

1. Study provided an adequate definition for control subjects in case-control study design: ^‡^

(1) Yes (0) No (0) Unclear

1. Rigor of cases sampling methodology: ^††^

(1) Probability-based

(0) Non-probability-based

(0) Unclear

1. Rigor of controls sampling methodology: ^‡^

(1) Probability-based

(0) Non-probability-based

(0) Unclear

**Quality category:**

Cohort & cross-sectional studies (9 quality criteria) Case-control studies (11quality criteria)

**Score of ≥7**: Potentially of low risk of bias (ROB) **Score of ≥9**: Potentially of low risk of bias (ROB)

**Score of <7**: Potentially of high ROB **Score of <9**: Potentially of high ROB

^*^ Applicable to all studies

^†^ Applicable to cohort and cross-sectional studies only

^‡^ Applicable to case-control studies only
