# Supplementary material for: Association between diabetes mellitus and active tuberculosis: A systematic review and meta-analysis
Source: PLoS One. 2017 Nov 21;12(11):e0187967. doi: 10.1371/journal.pone.0187967 (PMC5697825; doi:10.1371/journal.pone.0187967)
Supplement: S1 Text — (DOCX) [file pone.0187967.s007.docx]

**S2 Text. Calculation of attributable risk fraction of TB among DM patients and population attributable risk fraction of TB due to diabetes**

Attributable risk fraction (ARF) [1]

$$ARF=\frac{\left(RR-1\right)}{RR}\times100\%=\frac{\left(3.59-1\right)}{3.59}\times100\%=72.1\%$$

where RR = relative risk (i.e. RR = 3.59 for overall strength of association based on the pooled estimate of prospective studies)

Population attributable risk fraction (PARF)[1]

$$PARF=\left\{\frac{P(E)\times\left(RR-1)\right)}{P(E)\times\left(RR-1\right)+1}\right\}\times100\%$$

where $P(E)$ is prevalence of exposure

**India**: DM prevalence of 8.7% [2] $PARF=\left\{\frac{0.087\times\left(3.59-1)\right)}{0.087\times\left(3.59-1\right)+1}\right\}\times100\%=18.4\%$

**Indonesia**: DM prevalence of 6.2% [2] $PARF=\left\{\frac{0.062\times\left(3.59-1)\right)}{0.062\times\left(3.59-1\right)+1}\right\}\times100\%=13.8\%$

**China**: DM prevalence of 10.6% [2] $PARF=\left\{\frac{0.106\times\left(3.59-1)\right)}{0.106\times\left(3.59-1\right)+1}\right\}\times100\%=21.5\%$

**Nigeria**: DM prevalence of 4.6% [3] $PARF=\left\{\frac{0.046\times\left(3.59-1)\right)}{0.046\times\left(3.59-1\right)+1}\right\}\times100\%=10.6\%$

**Pakistan**: DM prevalence of 6.9% [2] $PARF=\left\{\frac{0.069\times\left(3.59-1)\right)}{0.069\times\left(3.59-1\right)+1}\right\}\times100\%=15.2\%$

**South Africa**: DM prevalence of 7.0% [2] $PARF=\left\{\frac{0.07\times\left(3.59-1)\right)}{0.07\times\left(3.59-1\right)+1}\right\}\times100\%=15.3\%$

**References**

1. Szklo M, Nieto FJ. Epidemiology: Beyond the Basics. Third ed. United States of America: Jones & Bartlett Learning; 3 edition; 2012 November 7, 2012.

2. International Diabetese Fedration. International Diabetese Fedration Atals 2015. <http://www.diabetesatlas.org/(accessed> September 15, 2016).

3. Isara AR, Okundia PO. The burden of hypertension and diabetes mellitus in rural communities in southern Nigeria. Pan Afr Med J. 2015;20:103. doi: 10.11604/pamj.2015.20.103.5619. PubMed PMID: 26090051; PubMed Central PMCID: PMCPMC4458303.
